# Antioxidative effects of phytoaromatic compounds on the cysteine of the SKCGS peptide as a critical aggregation domain of tau

**DOI:** 10.1038/s41598-025-04999-x

**Published:** 2025-07-02

**Authors:** Afsaneh Roshanfekr, Zahra Moeini, Farhad Golmohammadi, Saeed Balalaie, Arefeh Seyedarabi

**Affiliations:** 1 Institute of Biochemistry and Biophysics, University of Tehran, Tehran, Iran; 2 Peptide Chemistry Research Group, K.N. Toosi University of Technology, PO Box 15785-4416, Tehran, Iran

**Keywords:** Oxidative stress, Disulfide bond, SKCGS peptide, Tau protein, Alzheimer’s disease, Biochemistry, Neuroscience, Plant sciences, Diseases

## Abstract

Cysteine residues play a crucial role in maintaining the structural and functional integrity of proteins, but they are highly susceptible to oxidative modifications. These modifications can contribute to disorders such as Alzheimer’s disease (AD). In tau protein, the SKCGS sequence, especially the cysteine residue at position C-322, is particularly vulnerable to oxidative stress (OS). This vulnerability promotes tau fibrillation and aggregation, both in vitro and in vivo. This study evaluated the antioxidative effects of cinnamaldehyde (Cin), phenylethyl alcohol (PEA), and the common crude spices (cinnamon, rose, saffron, and cardamom) on the SKCGS peptide of the tau protein (in solution and aroma forms), with a focus on their ability to prevent oxidative damage. We adopted a novel approach using aromatherapy that utilizes the aromatic and volatile properties of natural compounds. By combining biochemical assessments with molecular docking studies, we aimed to provide a comprehensive understanding of the protective mechanisms of the natural phytoaromatic compounds used in this study. The antioxidative potential of these compounds was assessed using the DPPH assay; thiol levels measured via DTNB and mBBr assays; and binding interactions evaluated through molecular docking. The DPPH assay confirmed the antioxidative activity of all tested compounds. DTNB and mBBr assays showed that the phytoaromatic compounds significantly preserved free thiol content in the SKCGS peptide under oxidative conditions (*p* < 0.05). Molecular docking predicted favorable binding interactions between the phytoaromatic compounds and the cysteine residue in the SKCGS sequence, suggesting a protective mechanism against OS. The data presented provided strong evidence supporting the idea that phytoaromatic compounds could be promising candidates for developing innovative treatment approaches for AD. The encouraging results from this study justify further exploration of phytoaromatic compounds as therapeutic agents for AD. This research opens up new avenues for creating effective treatments to address one of the most significant challenges in neurodegenerative medicine.

**Supplementary Information:**

The online version contains supplementary material available at 10.1038/s41598-025-04999-x.

## Introduction

Thiol groups, whether present in small molecules, peptides, or proteins, are highly reactive and susceptible to oxidation by various spontaneous oxidative post-translational modifications (oxPTMs) and can be oxidized, resulting in changes in protein structure and function^1–3^. The free sulfhydryl group can reversibly adopt many oxidation states including disulfide bonds (–S–S–) and sulfenic acid (–SOOH). Cysteine is found in relatively few proteins (2% of all residues in proteins are cysteine), but due to its chemical diversity, it can significantly determine the structure and function of proteins^4–6^. The majority of protein thiols have a pK_a_ of 8–9, which makes them largely protonated at physiological pH. However, reactivity is not only determined by pK_a_; the protein’s microenvironment, including variations in polarity and charge can also play a role . The oxidation state of sulfenic acid is typically unstable and short-lived^1^. The in vitro formation of disulfide bonds may occur spontaneously between two thiolate anions, with molecular oxygen serving as the final electron acceptor. The final oxidant in many pathways for disulfide bond formation is molecular oxygen. The direct reaction between triplet dioxygen and singlet thiol is spin-forbidden, leading to a slow process that primarily causes hydrogen peroxide^7^. Hydrogen peroxide can promote a chain reaction with the reversible formation of sulfenic acid and then a disulfide bond. The findings suggest that abnormal levels of disulfide bonds are closely linked to various health disorders, including tau protein accumulation in tauopathies^8^.

**Fig. 1:**
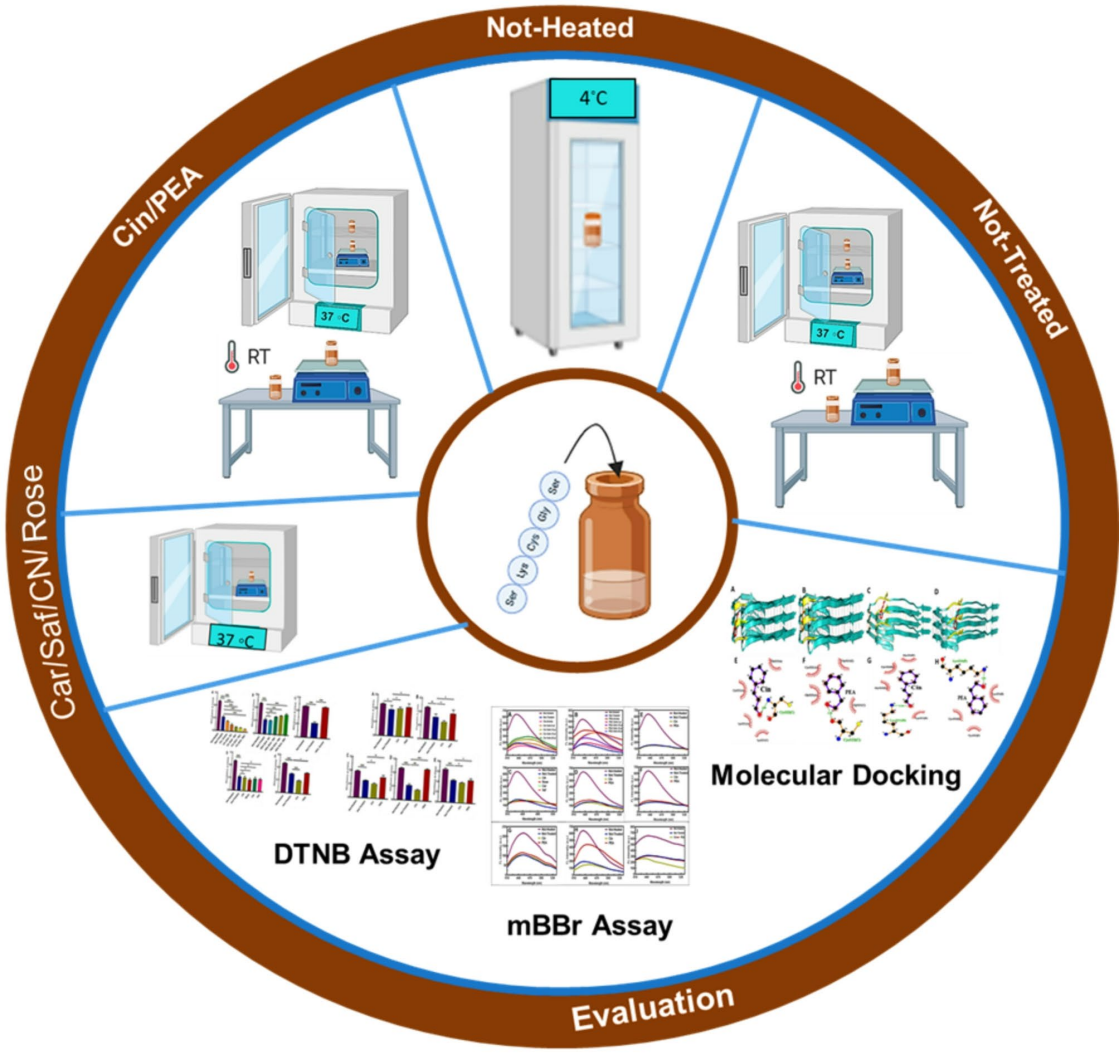
Schematic illustration of the in vitro and in silico studies conducted on the antioxidative effects of phytoaromatic compounds on the cysteine of the SKCGS peptide, as a critical aggregation domain of tau protein (figure made using the BioRender website).

Tau is a microtubule-associated protein predominantly expressed in the central and peripheral nervous systems, where it stabilizes microtubules by binding to tubulin through R2 and R3 repeats containing the SKCGS domain, playing a vital role in maintaining neuronal morphology, axonal development, and navigation^9^. Under normal physiological conditions, wild-type tau is highly soluble and resistant to aggregation^10^. Tau protein consists of cysteine residues and the oxidation of one of these residues, C-322 in the SKCGS sequence (found in the R3 repeat of tau protein), appears to promote tau aggregation into paired helical filaments (PHFs)^11^. Tau aggregates are observed not only in AD but also in other neurodegenerative tauopathies such as Pick’s disease (PiD) and progressive supranuclear palsy (PSP). These disorders are characterized by abnormal tau accumulation within neurons, leading to neuronal dysfunction^12^. AD is recognized as a neurodegenerative tauopathy characterized by the accumulation of the microtubule-associated protein tau into unstable aggregates known as neurofibrillary tangles (NFTs)^13^. Many bioactive compounds and natural plant extracts have been explored for potential treatments of dementia and AD. While AD is a complex disorder, oxidative stress (OS) significantly contributes to the damage in cholinergic neurons. Antioxidant mechanisms targeting OS fall into three categories: (1) inhibition of free radical formation (indirect antioxidants), (2) direct scavenging of free radicals (direct antioxidants), and (3) enhancing the cell’s ability to manage elevated reactive oxygen species (ROS) levels (metabolic antioxidants). Most bioactive compounds primarily act as direct antioxidants, neutralizing free radicals independently of cellular enzymes. The rising interest in phytochemicals is likely due to their low toxicity and synergistic effects. Additionally, bioavailability is crucial, as it determines the proportion of a compound that reaches its target site after administration^14^. The protection of sulfhydryl groups in proteins from oxidation depends on the antioxidant capacity^2^. Phenolic compounds, including flavonoids, and polyphenolic compounds, are amongst the secondary metabolites found widely in plants and human diet^6^. Phenylethyl alcohol (PEA) from *Rosa Damascena* and Cinnamaldehyde (Cin) from *Cinnamomum cassia,* as potent aromatic antioxidants can protect biomolecular structures such as proteins from oxidative damage^15,16^. Cin, an active compound found in Cinnamon, helps maintain cellular redox balance by inhibiting the production of ROS. Despite its low bioavailability of 20%, Cin remains in the blood of experimental animals as a metabolite for an extended period^17,18^. For instance, studies have shown that Cin can effectively enhance the activities of superoxide dismutase (SOD) and catalase, two important antioxidant enzymes^19^. According to our previous findings, Cin and PEA play a role in preventing tau fibril formation, and data have shown that PEA is effective in significantly reducing the formation of these fibrils. These compounds can be used in aromatherapy due to their volatile and aromatic properties^20^. Having a low molecular weight, being non-polar, and having antioxidant properties, even at low concentrations, makes aromatic active constituents such as PEA and Cin, effective in preventing the accumulation of proteins in the brain, through their volatile nature, without involving complications of the blood-brain barrier (BBB). DPPH is the most common *in* *vitro* method used to evaluate the antioxidant activity of phenolic compounds. It is rapid and simple as it does not involve many steps and reagents, and thus is inexpensive compared to other test models^21^. In the current study, we have evaluated the antioxidant effects of pure active constituents Cin, PEA, as well as common crude spices such as Cinnamon (CN), Rose, Saffron (Saf), and True Cardamom (Car) on the SKCGS tau peptide. The selection of these compounds was based on their widespread dietary use, established safety profiles, and previous evidence of biological activity, making them suitable candidates for further investigation. For example, Cin is a major component of cinnamon, a spice extensively used worldwide, with demonstrated antioxidant and anti-inflammatory properties comparable to other polyphenols like quercetin or epigallocatechin gallate (EGCG), but with the advantage of natural dietary inclusion and low toxicity^22,23^. Similarly, PEA is naturally present in Rose oil, with documented antioxidant effects that surpass synthetic antioxidants like Trolox, which can have safety concerns with chronic use. Comparing to other bioactive natural compounds, these spices and constituents are more accessible, have a long history of safe consumption, and are already accepted in food and industries, facilitating translation into potential therapeutic applications. Moreover, their volatile nature and low molecular weight enable rapid absorption and diffusion, unlike larger or more complex molecules such as curcumin or resveratrol, which often suffer from poor bioavailability^24,25^. Therefore, their selection is justified not only based on traditional use but also because they possess favorable pharmacokinetic properties and documented bioactivity, making them promising, cost-effective agents for neuroprotection and antioxidant therapy. This study aimed to investigate the protective effects of phytoaromatic antioxidant compounds on the reduced form of cysteine in the SKCGS peptide, which is a hotspot for the initiation of tau protein nucleation under OS conditions. These phytoaromatic antioxidants protect thiol groups in the SKCGS peptide from oxidative damage and are expected to interact with cysteine residues, influencing their oxidation state. Focusing on this short peptide will enable the direct investigation of the biochemical changes caused by oxidation, avoiding the complications that come with full-length tau^26^ or larger fragments, which may introduce additional interactions or modifications that obscure the role of the SKCGS motif in aggregation. The effect and binding mechanism of these compounds with cysteine ​​in the SKCGS peptide were analyzed using fluorescence spectroscopy, UV-Vis spectroscopy, and molecular docking (Fig. 1). In silico tools such as molecular docking can help predict ligand to protein interactions^27–30^. Therefore, in this study, additional to in vitro studies, we conducted molecular docking studies on the interaction between the phytoaromatic compounds and that of the SKCGS peptide sequence of the tau protein.

## Materials and methods

### Materials

Total Antioxidant Capacity (TAC) Assay Kit Based on DPPH Assay was obtained from the ZANTOX company, Iran. Common crude spices were purchased from Iran. Hydrochloric acid, Sodium chloride, and dithiothreitol (DTT) were obtained from MERCK. 5,5′-dithiobis(2-nitrobenzoic acid) (DTNB), Monobromobimane (mBBr), Tris (Hydroxymethylaminomethane), *trans*-cinnamaldehyde (Cin; Lot number MKBV8774V), and phenyl ethyl alcohol (PEA; catalog number W285803) and other chemicals were obtained from Sigma-Aldrich.

### Method

#### DPPH assay

The antioxidant activity of CN, Rose, Saf, Car, Cin, and PEA were evaluated three times. The common crude spices and the pure active constituents were dissolved in 50% ethanol, were mixed with DPPH solution and the resulting mixture was incubated in the dark at room temperature for 40 min. The Hybrid Multi-Mode Microplate Reader (SYNERGY™ H4, BIOTEK, USA) recorded the solution’s absorbance at 517 nm. The kit contained Trolox (TRO), which was dissolved with deionized water to form a control standard for pure active constituents. Green Tea (GT) was used as the standard control solution for common crude spices. The percentage of inhibition ratio was calculated using Eq. (1):

1 $$DPPH\;inhibition\;ratio{\text{ }}\left( \% \right){\text{ }}={\text{ }}\left( {{A_{DPPH}} - {A_{Sample}}/{A_{Sample}}} \right)*100.$$

A_DPPH_: represents the absorbance of the blank solution before the antioxidant is added.

#### Treatment of SKCGS peptide with common crude spices and pure active constituents

The SKCGS peptide solution (1 mM) was treated with 1, 5, and 10 mM DTT using different methods in the presence and absence of each compound, either the common crude spices (CN, Rose, Saf, Car) in the aroma form or the pure active constituents (Cin, PEA) in the aroma and solution forms. All solutions were prepared in 50 mM Tris buffer pH 6.8 containing 100 mM NaCl at a final volume of 2 to 2.20 ml based on the treatments either containing the aroma or solution forms of the compounds. For evaluation in the aroma form, 5 mg/ml of each common crude spice and 20 µl of the pure active constituents were transferred to a 1.5 ml microtube with 7 small holes, which were then placed inside bottles containing the SKCGS peptide solution  (Fig. S1 ). Therefore, the experimental set-up was such that , the bottles containing SKCGS peptide with or without the microtubes, were subjected to either shake at 500 rpm or no shake conditions for 24 h, at either 37 °C or RT. For each sample, the blank solutions lacking SKCGS peptide were incubated with aroma-producing compounds in aroma and in solution forms. Furthermore, the control SKCGS peptide sample (Not-Heated (NH) sample), lacking the microtube containing aroma-producing compounds  , was incubated at 4 ˚C for 24 h (Table S1 and S2).

#### DTNB assay

The DTNB assay was used to measure free thiol levels of SKCGS peptide samples in the presence and absence of aroma-producing compounds. DTNB in 0.1 M phosphate buffer (pH 7.2) was added to SKCGS peptide solutions at a final volume of 200 µl. The final DTNB concentration in all samples was 10 mM with SKCGS peptide concentration at 0.5 mM. The formation of thionitrobenzoic acid (TNB) was monitored at 412 nm after incubation at room temperature for 60–90 min using a Nano Pure infinity (BARNSTEAD).

#### mBBr assay

The mBBr fluorescence assay was performed to detect the free thiol contents of SKCGS peptide samples in the presence and absence of aroma-producing compounds. A fresh stock solution of mBBr (10 mM) was prepared in DMSO. Then, the excitation wavelength was fixed at 380 nm and the emission spectra was obtained at 450 nm. The mBBr was added to the SKCGS peptide (0.8 mM) to yield a peptide to mBBr ratio of 10:1 and incubated 60–90 min in the dark at room temperature. Moreover, the excitation/emission slit width were set at 10/20 nm.

#### Statistical analysis

The data was analyzed by GraphPad Prism version 6.0. One-way ANOVA followed by Dunnett’s multiple comparisons test was used to compare the differences observable between the different post-incubated samples and the controls. All the experiments were performed in triplicates.

#### Molecular docking analysis

Molecular docking was performed using UCSF Chimera software to predict the maximum binding affinity between ligands and protein crystal structures. LigPlot^+^ software was used to find the type of amino acid residue interaction with the ligand. Cin, PEA, CN (CNMA, Cin), Rose (PEA, Cit, Gra), Car (α-TA, CNL), and Saf (Safr, 2-B40, iBuCHO) were docked with the 3D structure of tau protein containing the SKCGS peptide sequence (PDB ID: 6HRE‌), which was obtained from the Protein Data Bank (PDB) (http://www.rcsb.org*).* In addition, utilizing the PubChem (https://pubchem.ncbi.nlm.nih.gov), the chemical structures of Cin, PEA, CN (CNMA, Cin), Rose (PEA, Cit, Gra), Car (α-TA, CNL), and Saf (Safr, 2-B40, iBuCHO) were obtained. Then, the protein structure had its polar hydrogen atoms added, followed by the detection of the ligand root after the removal of water molecules. To find the possible binding site of Ligands on the 3D structure of tau protein, a defined docking was done. Calculation and display of docking results were done using an online server https://www.dockthor.lncc.br/v2/ , Ligplot version 2.2.5, and Chimera version 1.15 software^31–33^. First, the chemical and three-dimensional structure of the volatile and aromatic extracts of Cin, PEA and the main volatile and aromatic compounds of CN, Rose, Saf, and Car were downloaded in SDF format from the https://pubchem.ncbi.nlm.nih.gov/. The SDF files were saved to PDB format using the Chimera software. Subsequently, the protein-ligand docking was done using the online DockThor server. To dock proteins and ligands using the free web server, first, the protein PDB file was uploaded followed by the protonated ligand PDB file. The user-defined molecular docking study was performed on the same possible binding pocket present in the tau protein to evaluate the binding behavior of all tested compounds in the same binding pocket and under identical conditions. We set the small grid box of docking covering the SKCGS structure of the tau protein. The size of the grid box was 25 × 22 × 17 Å, and the center of the grid center was 155.319 × 142,191 × 152.634 Å in the x, y, and z directions. This process allowed for efficient docking of the ligand onto the protein. Then, the best ligand conformer obtained from the docking job was converted to PDB format by the Chimera software as best ranking.mol2 and added to the protein PDB structure. Finally, the type of the ligand-protein interaction were observed and analyzed using the Ligplot software.

## Results and discussion

### Evaluation of antioxidant activity by DPPH assay

**Fig. 2:**
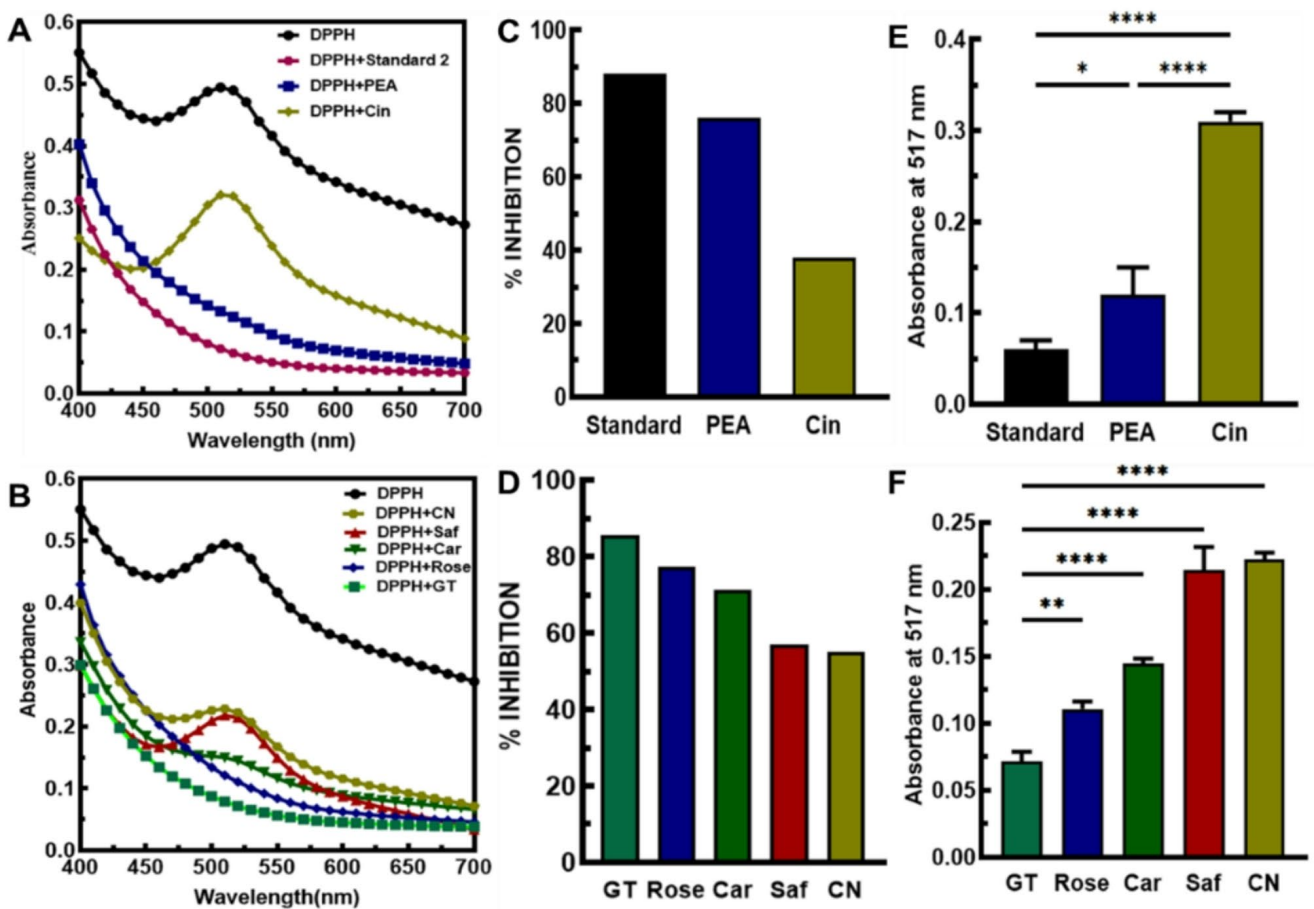
Evaluation of the phytoaromatic compounds’ antioxidant activity using DPPH in 50% ethanol. (**A**, **B**) The antioxidant activity results of pure active constituents and common crude spices, respectively, compared to standard solutions. (**C**, **D**) DPPH Inhibition percentage of phenolic compounds. (**E**, **F**) ANOVA test of phenolic compounds at 517 nm (*n* = 3). ANOVA test results: Significance level obtained at (*p* < 0.05).

In our study, we evaluated the antioxidant effects of various phenolic compounds using the proposed DPPH assay. The assay uses a stable free radical called α,α-diphenyl-β-picrylhydrazyl (DPPH; C18H12N5O6, M = 394.33)^34^ and measures the ability of antioxidants to scavenge DPPH by reducing the odd electron of the nitrogen atom to the corresponding hydrazine, through the donation of a hydrogen atom^35^. The antioxidant activity of phenolic compounds depends on their structure, especially the benzene ring and the number and position of the OH group. The benzene ring is responsible for stabilizing antioxidant molecules after reacting with free radicals^36^. The solubility and antioxidant activity of phenolic compounds depend on solvent polarity. A water/alcohol mixture increases the rate of their activity^37^. Based on our experimental results, PEA and Rose had the highest antioxidant capacity among the phenolic compounds. This was followed by Car, Saf, CN, and Cin, respectively (Fig. 1). The details of Fig. 2 are shown in Table S3 and S4. Rose comprises of 90.2% PEA^38^. Due to the hydroxyl group and benzene ring, PEA’s antioxidant stability and activity increase in pure form and as part of the crude form in Rose. PEA acts as a free radical scavenger by donating hydrogen atoms or electrons to ROS such as hydroxyl radicals (•OH) and superoxide anions (O2•−). This interaction effectively neutralizes the harmful effects of ROS.

### Simulation of oxidative stress

**Fig. 3:**
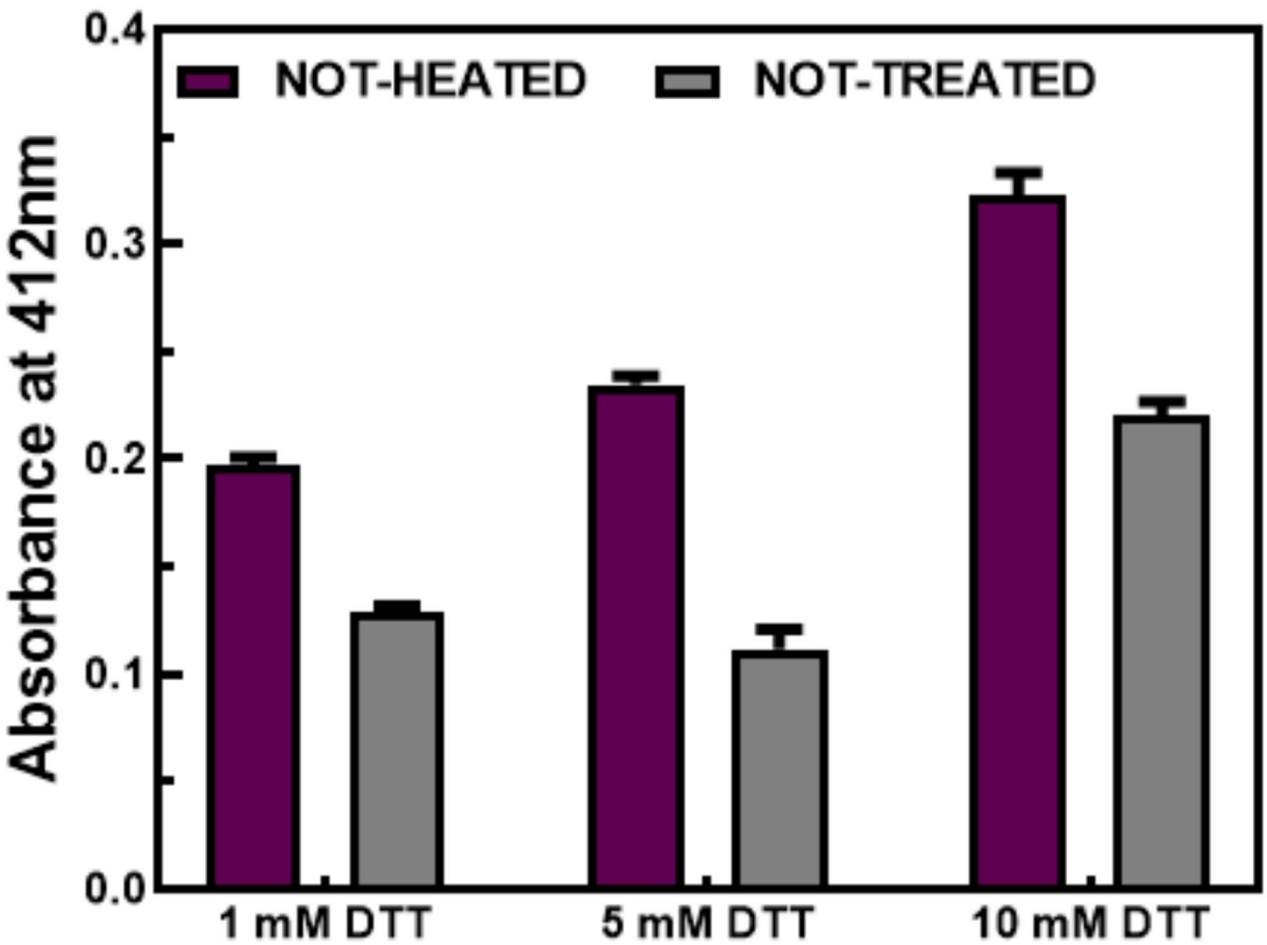
The effect of different DTT concentrations (1, 5, and 10 mM) on disulfide bond formation of SKCGS peptide at 37 °C with shaking.

Measurement of SKCGS peptide purity using HPLC and molecular weight using MALDI-TOF-MS was performed (High-resolution mass spectrum (HRMS) (ESI-TOF) was recorded by using Waters LCT Premier^™^ XE mass spectrometer) (Fig. S2). In this study, before evaluating the effects of OS under different conditions, we optimized the appropriate concentration of DTT. DTT is the most effective mercaptan and plays a crucial role in reducing thiols. The effect of DTT on protein structure and activity depends on its concentration and the number of free cysteines and disulfide bonds in the proteins^39^. The process of disulfide bond reduction with DTT is slow and may take several hours to complete^40^. Figure 3 reveals the use of three different concentrations of DTT (1, 5, and 10 mM) to maintain the reduction of thiols and disulfide bond formation under induced oxidative conditions using the DTNB assay. At a concentration of 5 mM DTT, the NH sample had a greater amount of free thiols than at a concentration of 1 mM DTT. Meanwhile, the NT sample at 5 mM DTT had a lower amount of free thiols compared to 1 mM DTT concentration. This indicates that increasing DTT leads to more thiol group reduction and consequently increased formation of disulfide bonds under OS conditions. The NH sample showed an increase in free thiols at 10 mM DTT concentration, while the NT sample showed fewer disulfide bond formation than expected. DTNB can react with DTT at a high rate^41^ and false results may occur due to the reaction between free thiols of DTT and DTNB caused by higher DTT values. Therefore, the use of DTT at different concentrations is a strategic approach to maintaining the appropriate thiol environment while ensuring that the assay conditions yield reliable and interpretable results. The optimal concentration of 5 mM DTT was chosen to maximize thiol reduction while minimizing erroneous interactions with the assay reagents.

**Fig. 4:**
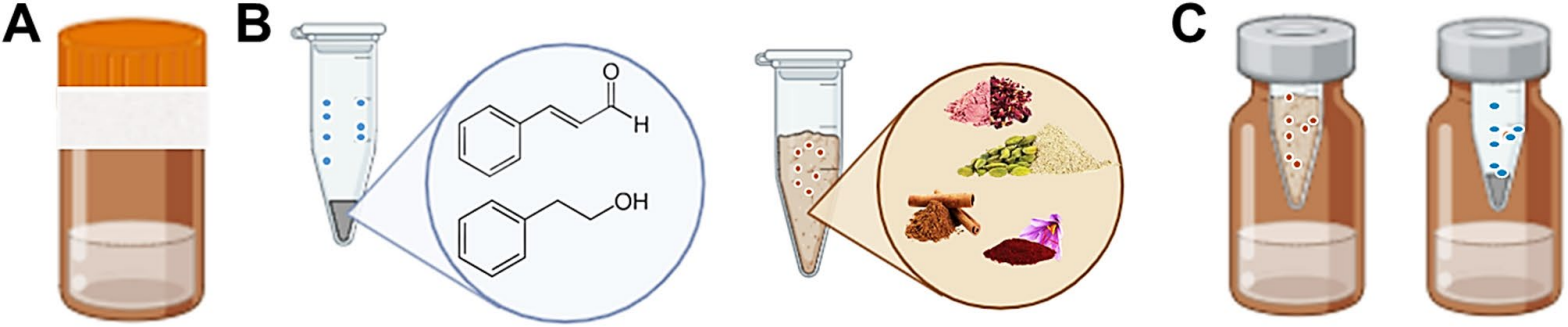
Experimental set-up. (**A**) SKCGS peptide solutions were placed in 20 ml bottles. (**B**) Holes were made in 1.5 ml microtubes, and then 20 µl of PEA, Cin, or 5 mg/ml of each common crude spices were added to the microtubes. (**C**) The microtubes were placed inside the 20 ml bottles and then sealed with aluminum foil and incubated under different conditions (figure was made using the BioRender website).

The OS was simulated in vitro: the thiol groups were oxidized, and then the nucleation phase of tau fibrils was monitored, which involved inter-molecular disulfide bond formation between the cysteines of the SKCGS peptides. The simulation process involved dark glass bottles, containing the SKCGS peptide solution, sealed with parafilm. This set-up caused oxygen to be trapped in the bottles, leading to different oxidation rates under different conditions (Tables S1, S2, and Fig. 4A). To create competitive conditions with oxidative agents and block or reduce their activities with aromatic antioxidants, the porous microtubes were placed inside each bottle containing the SKCGS peptide solution (Fig. 4B,C).

**Fig. 5:**
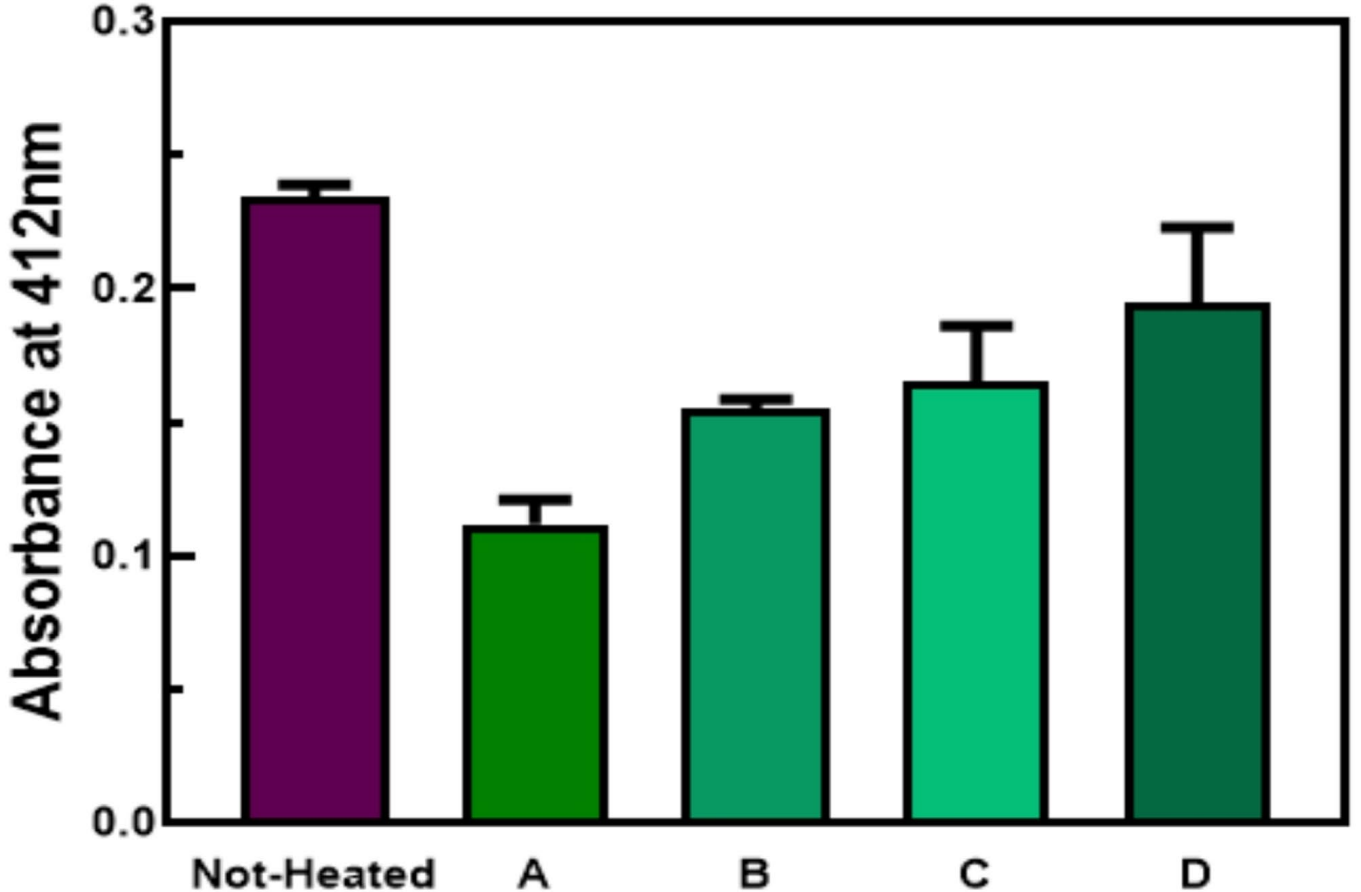
The effect of oxidative stress on the SKCGS peptide after 24 h of incubation. (**A**) At 37 °C with shaking. (**B**) At 37 °C without shaking. (**C**) At RT with shaking. (**D**) At RT without shaking.

Bottles containing solutions of SKCGS peptides were prepared and then divided into two groups and subjected to different temperatures. The first group was maintained at 4 °C to preserve the native structure of the SKCGS peptide and referred to as Not-Heated (NH) samples. The second group was maintained at 37 °C, shaken, and referred to as Not-Treated (NT) samples. In this study, we added DTT to reduce cysteines and investigated the impact of OS on cysteine as a factor affecting tau protein aggregation and fibrillation *in* *vitro* under different conditions. It is known that when the body is under OS, cysteines may get oxidized and converted to cysteine sulfenic acid (Cys-SOH). Sulfenic acids are unstable oxidation intermediates that can cause tau protein aggregation and fibrillation through the formation of disulfide bonds^42^. These cysteine residues contribute to the formation of tau’s toxic intermediate structures, including dimers and granular oligomers^43^. Our observations in various conditions demonstrated that temperature^44^ and shaking, increased OS, leading to cysteine oxidation and formation of disulfide bonds, compared to other conditions. Shaking imparts kinetic energy to the system, enabling molecules to surpass energy barriers and facilitate the formation of disulfide bonds more easily^45^. Temperature significantly influences the kinetics of disulfide bond formation. Typically, elevated temperatures enhance the rate of this process by providing more kinetic energy, thereby promoting molecular interactions. Using the DTNB colorimetric method (Fig. 5) revealed how the cysteine residue was affected by OS under different conditions in the NT samples relative to the NH sample. Based on the results, the NT sample A showed increased OS and formed more disulfide bonds. The formation of disulfide bonds in samples B and C were nearly the same. However, the results indicated that the impact of temperature alone has a greater effect on creating OS and disulfide bonds than the shaking effect alone. The NT sample D demonstrates that the rate of OS and disulfide formation is low under normal conditions. Our findings reveal that elevated temperatures and shaking amplify OS and foster disulfide bond formation in SKCGS peptide, with temperature having the most significant influence. This happens because higher temperatures boost molecular kinetic energy, accelerating cysteine oxidation and potentially paving the way for full-length tau fibril nucleation through disulfide cross-linking.

### Quantification of thiols and disulfides

#### Detection of thiols using DTNB colorimetry assay

**Fig. 6:**
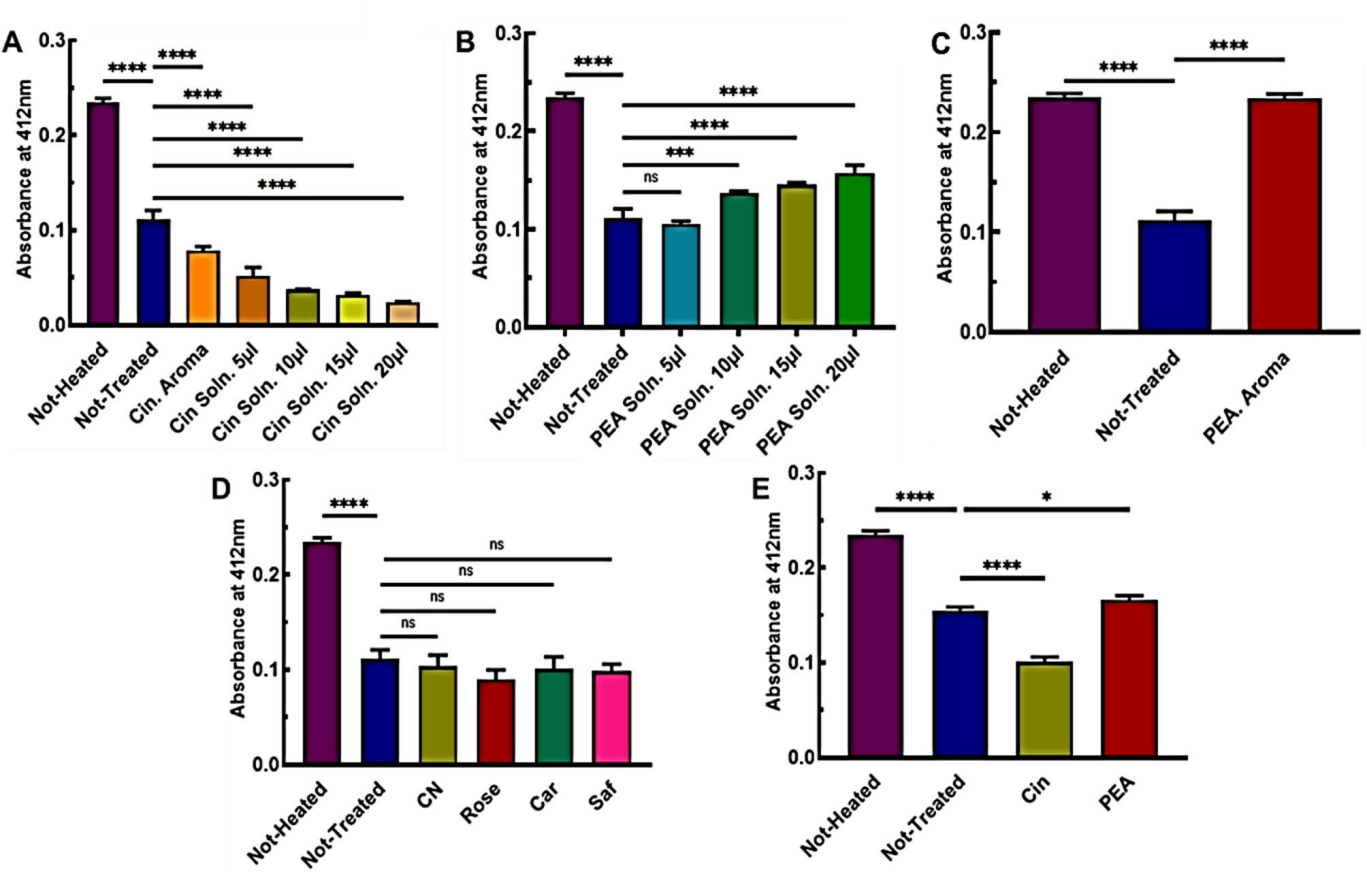
Detection of free thiols in the SKCGS peptide after 24 h of incubation at 37 °C with Cin and PEA in aroma and solution forms, as well as crude spices. (**A**–**C**) Aroma and solution forms of Cin and PEA at 37 °C, with shaking. (**D**) Common crude spices at 37 °C, with shaking. (**E**) Cin and PEA in aroma forms at 37 °C, without shaking.

The DTNB assay, also known as Ellman’s reagent, has been widely accepted and routinely used in different labs since its initial development by Arne Holmgren in 1977^46^. TNB⁻ is a vibrant yellow-colored ion that is generated through a disulfide exchange reaction. This process occurs when DTNB interacts with a free thiol group (–SH). DTNB was chosen as a widely used colorimetric reagent for selectively detecting thiols under different conditions. To evaluate the efficacy of antioxidants in thiol reduction maintenance and reducing disulfides, the DTNB reduction to two molecules of TNB that absorb at 412 nm was analyzed. In Fig. 6A-C, the antioxidant effects of Cin and PEA in aroma and in solution forms have been evaluated and compared. Cin has a non-polar structure and dissolves slowly in water at approximately 1.1 g/L at 20 ℃^47^. With the increase in volume of the Cin solution, the pH of the environment decreased according to Fig. 6A. The Ellman’s reagent has low sensitivity to detect free cysteines in acidic pH ranges because nucleophilic reaction can not occur efficiently^48^ and detects less free thiols in the aroma form of Cin (Fig. 6A), which slightly alters the pH of the environment. It is important to note that this reaction does not take place under acidic pH conditions, emphasizing the role of pH in the reactivity of thiol compounds. When Cin is added to the solution, the pH of the blank also decreases. This indicates Cin has entered into the solution. We observed that DTNB did not react with the peptide-containing solution incubated with the Cin solution. Additionally, we evaluated DTNB with the blank solution incubated with Cin as a control. The results of the colorimetric method are shown in Fig. S3. The research findings indicate that Cin can bind reversibly with free thiols, and these studies indicated that Cin failed to inhibit tau protein aggregation when the corresponding cysteine residues were mutated in the gene sequence of tau, and that Cin can only bind to cysteines only after 24 h of incubation ^49–51^. This could be another reason for the reduced detection of free thiols in aroma and solution forms, as Cin binds with the thiol of the cysteine residue, making it unrecognizable by DTNB. In Fig. 6B and 6C, PEA in its aroma form revealed a more significant effect in maintaining the reduced state of thiols than in its solution form. Also, the pH changes of the solution after incubation with PEA in the aroma and the solution forms, showed no noticeable difference. The results showed that more thiols are reduced by increasing the volume of PEA. Figure 6D showed that common crude spices do not have significant antioxidant effects in protecting the reduced state of thiols. Most of the antioxidant compounds have low solubility in aqueous solvents, but they showed significant antioxidant activity in water/alcohol solvents, as shown in Fig. 2B, 2D, and 2F. The solubility of insoluble compounds increases as the temperature rises^52^. It is shown in Fig. 6E that at 37 °C and without shaking, Cin and PEA in the aroma form, caused a decrease and slight increase in the amount of free thiols, respectively. According to the findings, raising the temperature resulted in an increase in the solubility of Cin in the aroma form, which led to the formation of bonds with the cysteine residue. Additionally, the amount of disulfide bonds was more in PEA at 37 °C with no shaking compared to PEA at 37 °C with shaking.

**Fig. 7:**
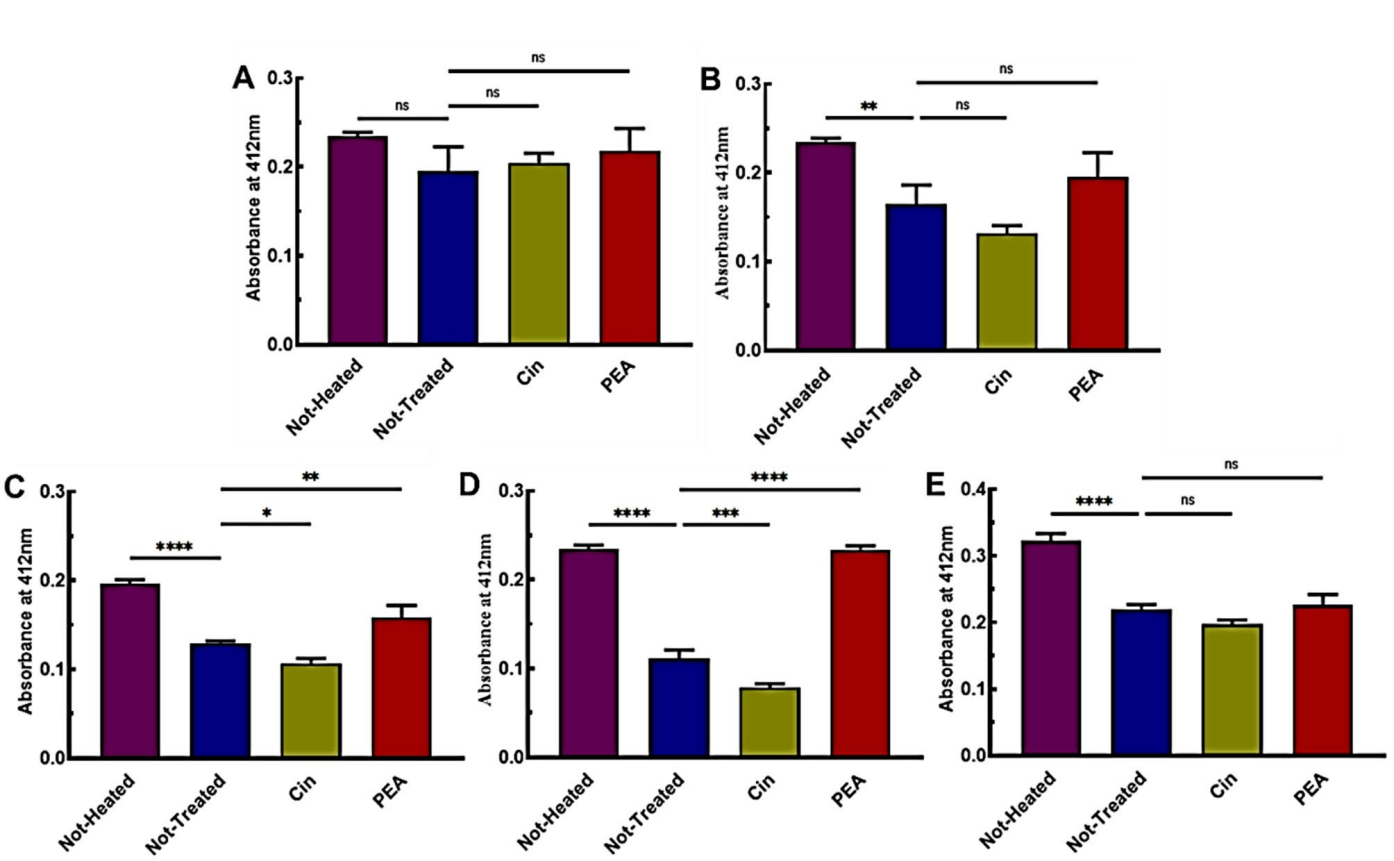
Detection of free thiols in the SKCGS peptide after 24 h of incubation with and without shaking, and at different DTT concentrations. (**A**, **B**) Free thiol detection for SKCGS peptide incubated with Cin and PEA in aroma form at RT, without shaking and with shaking, respectively. (**C**–**E**) 1, 5, and 10 mM DTT, respectively at 37 °C with shaking.

Based on the results, we concluded that Cin and PEA in aroma forms, better maintained the reduced state of free thiols at 37 °C and shaking conditions. Therefore, we incubated the samples with Cin and PEA in aromatic forms for 24 h at RT to evaluate the effects of temperature and shaking, separately. Figure 7A showed that the antioxidant activities of Cin and PEA were lower without shaking compared to shaking (Fig. 7B). Figure 7C, 7D and 7E revealed the effects of Cin and PEA at different concentrations of DTT. Cin and PEA exhibited comparable activities at 1 and 5 mM DTT, but no significant difference was observed compared to the NT sample at 10 mM DTT. We hypothesize that the presence of high amounts of DTT caused the detection of high levels of free thiols in Fig. 7E as a result of the reaction between DTNB and the free thiols present in the environment.

#### Fluorometry analysis by mBBr

**Fig. 8:**
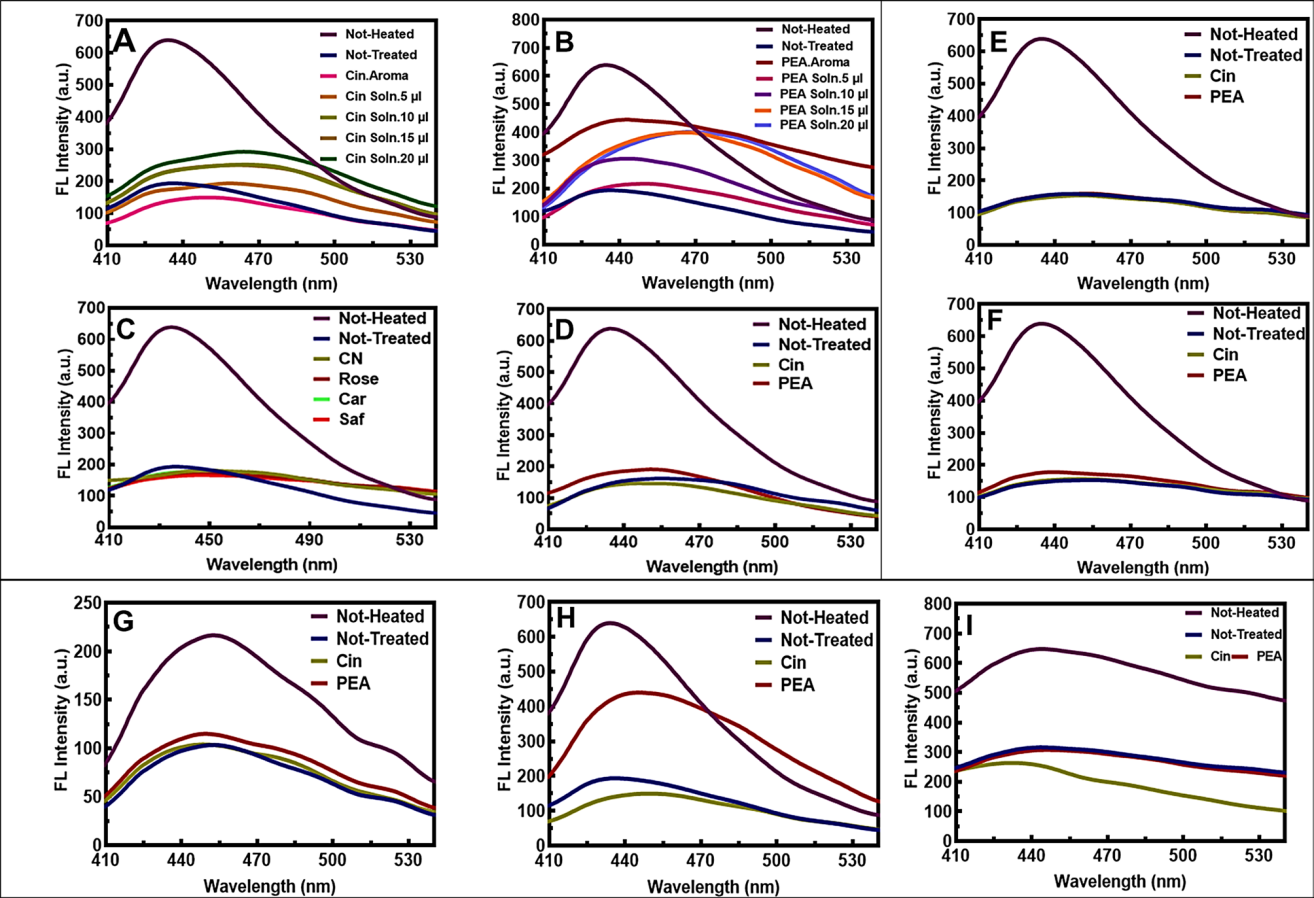
An mBBr-based assay for measuring free thiols in the SKCGS peptide after 24 h of incubation. (**A** and **B**) Aroma and in solution forms of Cin and PEA at 37 °C with shaking. (**C**) Common crude spices at 37 °C with shaking. (**D**) Cin and PEA in aroma form at 37 °C without shaking. (**E** and **F**) Cin and PEA at RT with and without shaking. (**G**-**I**) 1, 5, and 10 mM DTT concentrations, respectively at 37 °C, with shaking.

mBBr is a widely used fluorophore for labeling thiols and as a thiol-sensitive fluorescent probe; it has been reported to measure bioavailable H2S by Newton, Fahey, and Togawa et al.^53,54^]. mBBr has been used to quantify and measure nucleophilicity and acidity of LMW thiols and analyze thiol-containing proteins ^55,56^. After reacting with thiol, mBBr forms a thiol-mBBr adduct whose fluorescence emission can be measured^54^. mBBr is more sensitive than DTNB^57^, and the studies of George Tsakraklides et al. revealed that mBBr adducts have high stability in acidic pH^58^. mBBr’s greater sensitivity in detecting thiols compared to DTNB can be attributed to its direct and rapid reaction mechanism, strong fluorescence response, stability of the resulting products, effectiveness in acidic conditions, and lower susceptibility to interference^59,60^. The intrinsic pK_a_ value of the free cysteine thiol-thiolate equilibrium in water is approximately 8.6^61^. Figure 8A shows that the detection rate of free thiols by mBBr is directly proportional to the increase in the volume of Cin in the solution. The presence of Cin in a reaction solution is associated with a decrease in pH, according to the cysteine ​​PK_a_ and the DTT reducing power range at pH > 7^62^, more free thiols were detected in contrast with the NT sample. When Cin reacts with oxygen, it undergoes oxidation resulting in the production of cinnamic acid, which is one of the oxidation products of Cin, due to the formation of peroxides^63^. Figure 8B reveals that by increasing the volume of PEA in the solution and also in the aroma form, more thiols are observed. PEA dissolves slowly in water at 20 °C and under shaking conditions (2ml per 100ml of H_2_O)^64^. According to the results, PEA has been able to have significant effects in preserving free thiols in solution at 37 °C and under shaking conditions. It seems that PEA has a dual effect due to its high volatile and aromatic properties when dissolved in water, which can also provide its aromatic properties in solution. The results indicate that PEA is dispersed in the environment like a gas particle in a model of a volatile aromatic particle dispersion system. Shaking and temperature are two important factors that influenced the distribution of aromatic particles of PEA. When shaken, the PEA becomes more dispersed. Additionally, with an increase in temperature, the vapor pressure of PEA increased, leading to the release of more volatile particles into the environment. We speculate that the significant effect of PEA in the aroma form is due to the concentration of aromatic and volatile antioxidant particles in the environment, which caused oxidant species to be produced in small amounts. The results in Fig. 8C indicated that the common crude spices dissolved in water did not exhibit antioxidant activity, and thus no changes were observed. In aqueous solutions, they tend to aggregate, precipitate, or remain trapped, preventing their interaction with ROS or free radicals. Without sufficient dissolution, they cannot donate electrons or hydrogen atoms to neutralize ROS, which is the primary mechanism of their antioxidant action. PEA performed better than Cin at 37 °C, without shaking due to the difference in vapor pressure, as shown in Fig. 8D. Shaking caused more aromatic particles to be spread in the environment due to the higher vapor pressure of PEA compared to Cin. In Fig. 8E and 8F, there are no noticeable differences in the results for with shaking and without shaking conditions. However, under shaking conditions, PEA performed slightly better than Cin. Figure 8G–I shows the results of three different concentrations of DTT. PEA showed a better effect at a concentration of 5 mM DTT.

### Molecular docking

The molecular docking of the SKCGS peptide sequence of tau protein with phytoaromatic compounds in this study was done using the cryo-electron microscopy determined structure of the paired helical filament from a sporadic Alzheimer’s disease brain (PDB ID 6HRE)^65^. Due to the lack of high-resolution structures for either the SKCGS peptide alone or the native isoform of tau containing the peptide sequence in the Protein Data Bank (PDB), we could not perform molecular docking directly on the experimentally studied soluble form. Therefore, the molecular docking experiment was conducted using the 6HRE structure containing the SKCGS sequence. This structure had a higher resolution (3.2 Å) and better quality criteria among the structures available in the Protein Data Bank, related to tau. While docking to a fibrillar template provides valuable insights into potential binding sites and interactions, it may not fully reflect the conformational dynamics and accessibility of residues in the monomeric or solution-phase state. Therefore, the docking results should be interpreted as a model for interaction under the constraints of the available structure. Looking at the structure, the lysine residue is located on the surface, while the cysteine ​​residue is buried inside the structure and plays a role in the disulfide bond formation between the strands. Tau protein fibrils are formed by beta sheets. Docking parameters were defined individually for each chain, and the SKCGS sequence acted as an aggregation or fibrillation inducer under the influence of OS. In the fibrillation process, the cysteine in the SKCGS sequence is oxidized, which leads to the formation of disulfide bonds, resulting in the alignment of beta sheets on top of each other. Due to the placement of the SKCGS sequence in two different positions and for the purpose of investigating the effects of certain compounds in reducing fibrillation and aggregation, the tau paired filament structurewas separated in two groups of chains refereed to as chains A, C, and E, and chains B, D, and F. Molecular docking was performed competitively using the main volatile and aromatic compounds found in common crude spices. The molecular docking analysis showed that Lys321, Cys322, Gly323, and Ser324 are key binding residues in the SKCGS peptide across all chains. They play a crucial role in stabilizing ligand-protein complexes through hydrophobic interactions and hydrogen bonding, significantly affecting peptide-ligand affinity (Tables S5–6 and Figs. S4–11). There are several types of interactions that occur between ligands and the protein, including hydrogen bonding, electrostatic interactions, hydrophobic interactions, and Van der Waals forces. When the binding energy is reduced, the binding affinity increases between the ligand and the protein. Common crude spices are made up of several volatile and aromatic compounds and hence the major common volatile compounds were selected for docking in this study (Fig. S12)^38,66–73^.

**Fig. 9:**
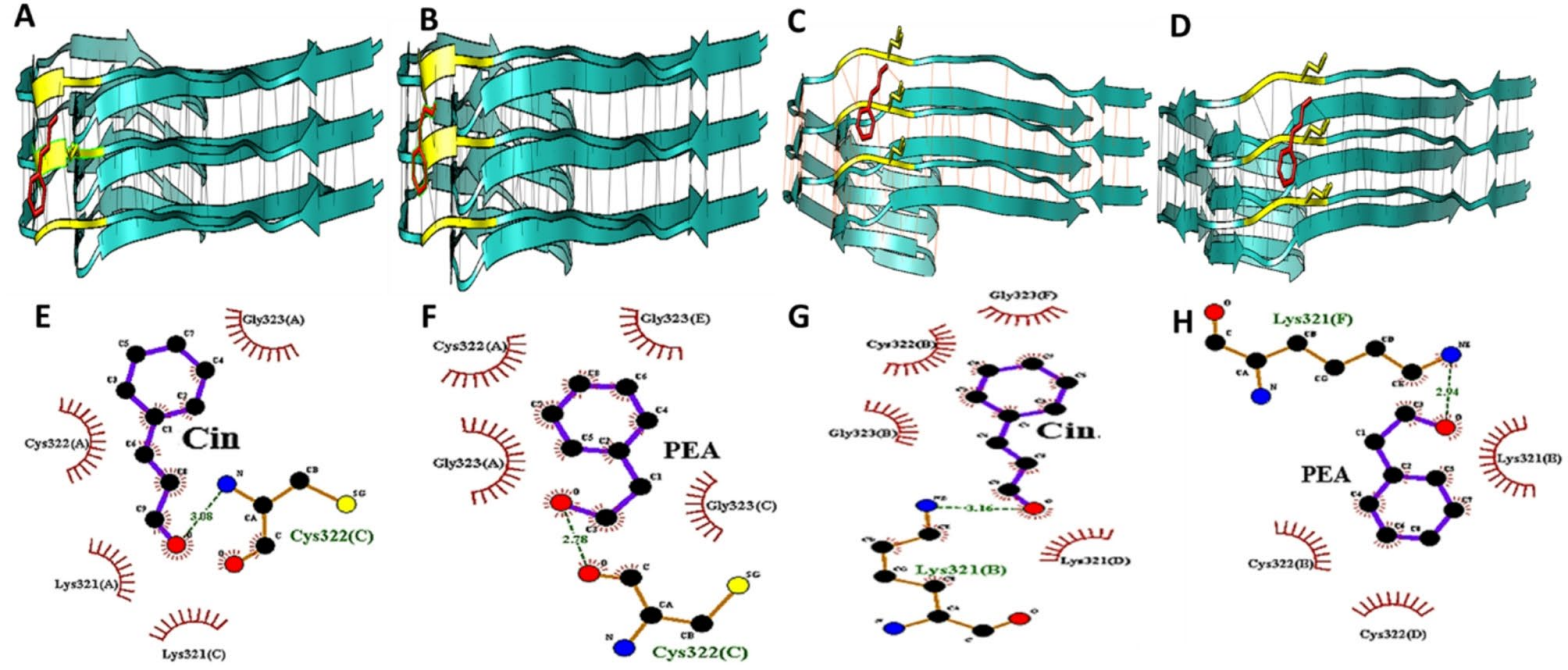
Molecular docking analysis of Cin and PEA binding to the SKCGS sequence of 6HRE. (**A**–**D**) The binding interactions of Cin and PEA with chains A, C, E, and B, D, and F, respectively, were visualized using Chimera. (**E**–**H**) The detailed interaction diagrams generated by LigPlot illustrate the binding of Cin and PEA to chains A, C, E, and B, D, F.

Table 1 indicates that Cin and PEA formed a hydrophobic bond with the cysteine in the SKCGS sequence in all chains. Cin and PEA formed hydrogen bonding with the cysteine in chains A, C, and E with equal energies. The result of the molecular docking of compounds with the SKCGS sequence of 6HRE was shown in Fig. 9.

**Table 1:** Binding affinity, hydrogen bonding and hydrophobic interactions between Cin, PEA, and the SKCGS sequence of 6HRE.

Active constituents	Chains used	Affinity (kcal/mol)	Hydrogen bonding		Hydrophobic interaction
			Amino acid (chain)	Distance (Å)	Amino acid (chain)
Cin	A, C, E	− 6.5	Cys322(C)	3.08	Cys 322(A) Lys 321(A, C) Gly 323(A)
	B, D, F	− 6.7	Lys 321(B)	3.16	Lys 321(D) Cys 322(B) Gly 323(B, F)
PEA	A, C, E	− 6.4	Cys322(C)	2.78	CYS 322(A) Gly323(A, C, E)
	B, D, F	− 6.7	Lys321(F)	2.94	Lys 321(B) CyS322(B, D)

**Fig. 10:**
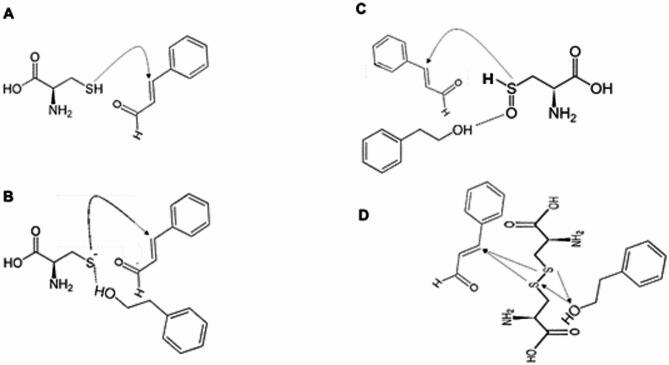
Chemical interactions of Cin and PEA with (**A**) thiol, (**B**) thiolate, (**C**) sulfenic acid, and (**D**) disulfide bond.

Based on the structures of Cin and PEA shown in Fig. S12, it is possible to speculate their binding mechanism to different states of the thiol group shown in Fig. 10.

**Fig. 11:**
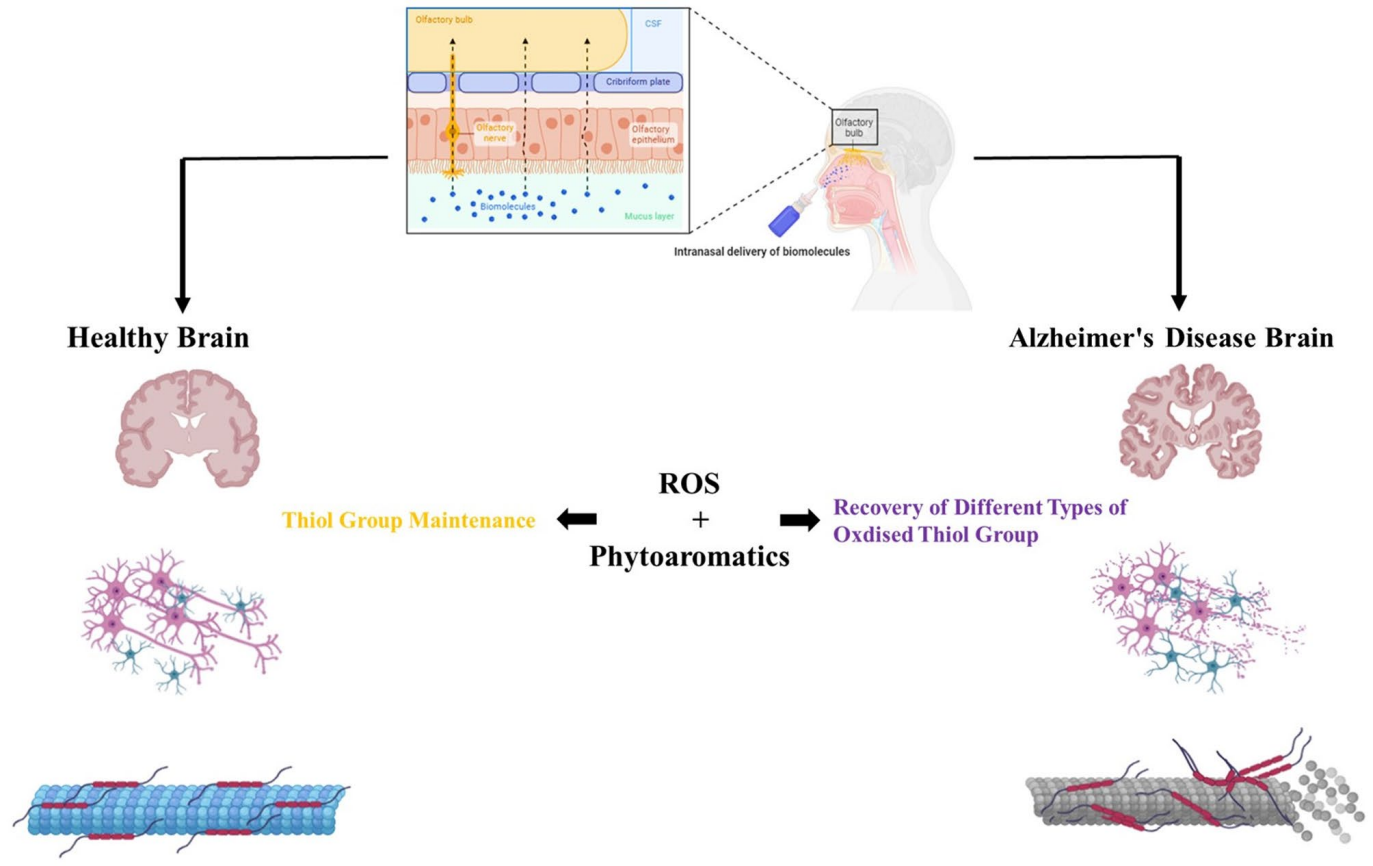
Schematic illustration of the antioxidative effects of phytoaromatic compounds on the healthy and Alzheimer’s disease brain (figure made using the BioRender website).

During oxidation, thiols can exist in four states: thiol, thiolate ion, sulfenic acid, and disulfide bond. Cin can react with thiol groups via nucleophilic Michael addition, where the thiol attacks the electrophilic carbon of Cin’s carbonyl group, forming a thioether linkage. This stabilizes the thiol group and prevents it from the oxidation process. PEA also acts as an antioxidant, scavenging free radicals, indirectly protecting thiol groups from oxidation and maintaining their reduced state. The thiolate anion state, which is more nucleophilic than the neutral thiol, readily reacts with Cin’s carbonyl carbon to form a thioether. This helps protect the thiolate from further oxidation, maintaining the protein’s functional state. Additionally, PEA stabilizes the thiolate anion by forming hydrogen bond interactions, reducing its reactivity and protecting it from further oxidation. Cin can react with sulfenic acid through nucleophilic addition or by forming a reversible adduct. This stabilizes sulfenic acid, preventing it from further oxidation to sulfinic or sulfonic acids, thereby averting irreversible oxidative damage. PEA can form hydrogen bonds with sulfenic acid, stabilizing it and preventing further oxidation to sulfinic or sulfonic acids. Cin can reduce disulfide bonds back to thiols, possibly via nucleophilic attack by a thiolate intermediate. This reduction reverses some oxidative modifications and potentially restores the protein function. PEA helps maintain a reducing environment through its antioxidant properties, indirectly supporting the reduction of disulfide bonds by forming the thioether bonding to thiols and protecting thiols and thiolates from oxidation, thereby preserving protein function. The aforementioned binding mechanisms demonstrate that Cin and PEA can prevent the side effects of oxidative stress by binding to the cysteine R group and maintaining the reaction environment (Fig. 11).

### Conclusions

Overall, OS plays a major role in neurodegenerative diseases by increasing oxidative processes, reducing antioxidant defenses, or both. Recent evidence suggests that OS may be the primary cause of neurodegenerative toxicity. The brain’s consumption of oxygen results in the production of free radicals, and its high oxygen requirement leads to an even greater number of ROS ^74^. Seeking antioxidants from plants, such as natural phenolic phytochemicals, seems to provide metabolic benefits and reduce the risk of developing several health problems ^75,76^. Aromatherapy is a nasal therapy system, which can deliver phytoaromatic antioxidants to the brain, bypassing the BBB. The key benefits of intranasal delivery include easy administration, fast onset of action, and the avoidance of gastrointestinal and hepatic first-pass effects. As a result, the nasal route is highly valuable for administering active substances in low doses with low oral bioavailability^77,78^. Intranasal delivery has emerged as a non-invasive method to bypass the BBB and deliver therapeutics directly to the brain through olfactory and trigeminal nerves innervating the nasal passages ^79^. This study offers valuable insights into the role of phytoaromatic antioxidants, but it also has notable limitations that should be considered. The in vitro findings may not directly translate into in vivo systems. The complex interactions present in a living organism, such as metabolic pathways, enzyme activities, and the influence of various cellular environments, can significantly alter the effects of the tested compounds. Therefore, caution should be exercised when extrapolating these results to clinical scenarios. Future research should involve testing these compounds in tau aggregation assays that utilize full-length tau protein and examining their effects in animal models. Future research should concentrate on using in vivo models, such as tau transgenic mice, to evaluate the effectiveness of these compounds in reducing tau pathology and enhancing cognitive function. Furthermore, investigating advanced delivery methods, like intranasal nanoemulsions, could improve the bioavailability of these compounds in the brain and increase their therapeutic potential.

The docking results showed that the phytochemical compounds analyzed in this study can potentially bind to the SKCGS peptide in the tau protein. While other techniques like Surface Plasmon Resonance (SPR) or Isothermal Titration Calorimetry (ITC) could provide more insights into binding interactions, the molecular docking findings helped enhance our understanding of how these compounds could interact with the SKCGS peptide, in order to provide protective effects. Future studies using other techniques can address more precise modes of interactions.

The significance of this study lies in its potential to identify novel therapeutic agents for AD, a condition characterized by oxidative stress and tau protein aggregation. These natural antioxidants were chosen based on their affordability, biocompatibility, safety, long-lasting shelf-life, ability to cross the olfactory pathway, and BBB permeability. By demonstrating the antioxidative effects of phytoaromatic compounds on the SKCGS peptide, a critical region involved in tau aggregation, this research contributes to the development of new strategies to combat neurodegeneration. Furthermore, the use of aromatherapy as a delivery method presents an innovative approach that could bypass the BBB, a major challenge in the treatment of neurological disorders.

## Electronic supplementary material

Below is the link to the electronic supplementary material.

Supplementary Material 1

## Data Availability

Data is provided within the manuscript or supplementary information files.
