# Supplementary material for: Antioxidative effects of phytoaromatic compounds on the cysteine of the SKCGS peptide as a critical aggregation domain of tau
Source: Sci Rep. 2025 Jul 2;15:23206. doi: 10.1038/s41598-025-04999-x (PMC12222454; doi:10.1038/s41598-025-04999-x)
Supplement: Supplementary file 1 — Supplementary Material 1 [file 41598_2025_4999_MOESM1_ESM.docx]

**Table S1. pH analysis of SKCGS peptide treated with aroma-producing compounds at 37 ℃.**

1. Before (B) After


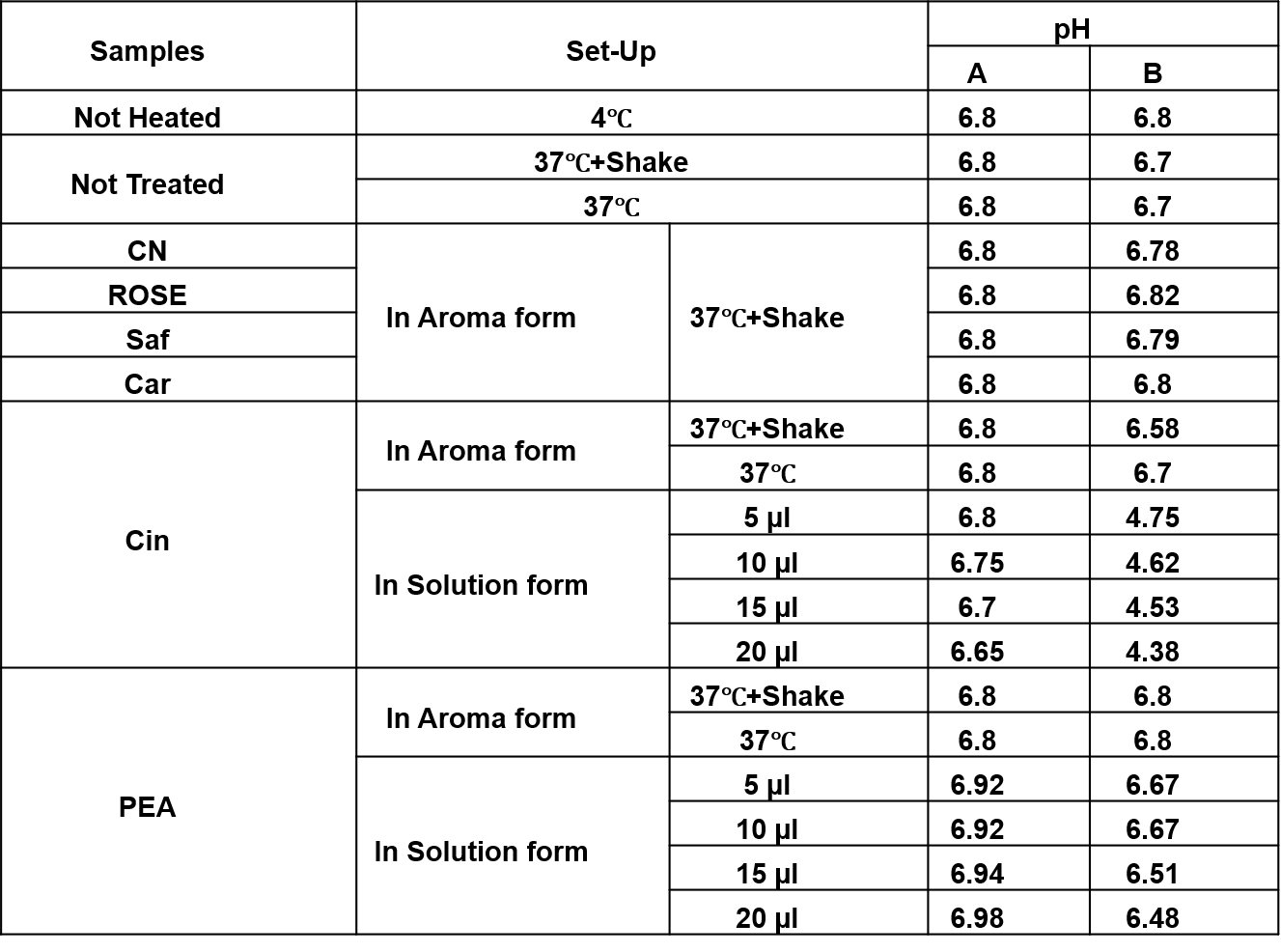


**Table S2. pH analysis of SKCGS peptide treated with aroma-producing compounds at RT.**


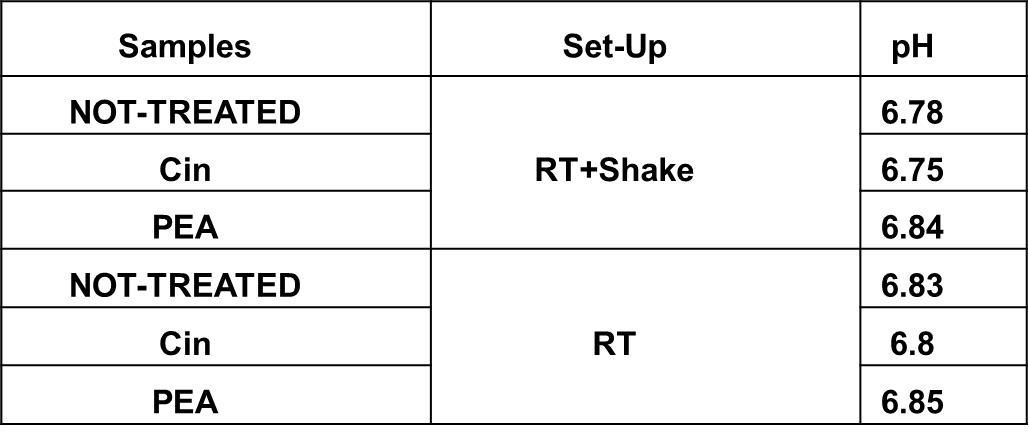


**Table S3. Antioxidant strength of the active constituents compared to Trolox.**


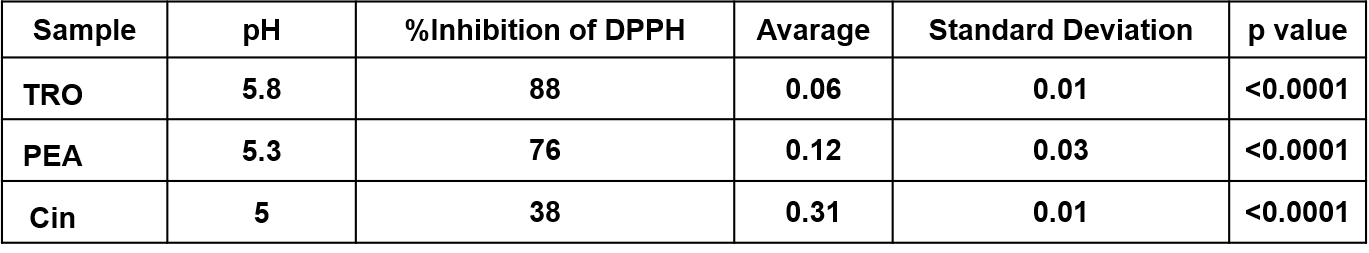


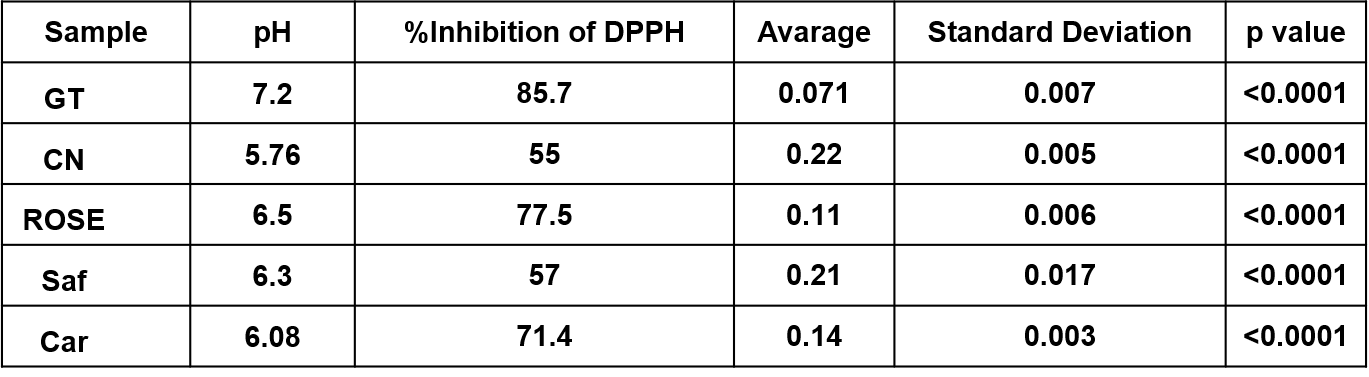


**Table S4. Antioxidant strength of the common spices compared to Green Tea.**

**Table S5. Binding affinity, hydrogen bonding, and hydrophobic interactions of ligands to the SKCGS sequence in chains A, C, and E.**

| **Hydrophobic interactions**  **Amino acid (chain)** | **Hydrogen Bonding** | | **Affinity**  **(kcal/mol)** | **Crude spices and their constituents** | |
| --- | --- | --- | --- | --- | --- |
|  | **Distance (Å)** | **Amino acid (chain)** |  |  |  |
| **Lys 321 (A,C)**  **Cys 322 (A)**  **Gly 323 (A)** | **2.83** | **Cys322(C)** | **-6.4** | **CNMA** | **CN** |
| **Lys 321 (A,C)**  **Cys 322 (A)**  **Gly 323 (A)** | **3** | **Cys322(C)** | **-6.5** | **Cin** |  |
| **Cys322(A,C,E)**  **Gly323(A.C.E)** | **-** | **-** | **-3.9** | **α-TA** | **Car** |
| **Cys322(A,C)**  **Gly323(A.C)**  **Ser 324 (A,C)** | **-** | **-** | **-6.3** | **CNL** |  |
| **Cys 322 (A)**  **Gly323(A.C.E)** | **2.87** | **Cys322(C)** | **-6.4** | **PEA** | **ROSE** |
| **Cys322(A,C,E)**  **Gly323(A.C.E)** | **-** | **-** | **-6.4** | **Cit** |  |
| **Cys 322 (A)**  **Gly323(A.C.E)**  **Ser 324 (C)** | **-** | **-** | **-6.4** | **Gra** |  |
| **Cys322(A,C)**  **Gly323(A.C)** | **-** | **-** | **-6.1** | **Safr** | **Saf** |
| **Lys 321 (A,C)**  **Cys 322 (A)**  **Gly 323 (A)** | **3.14** | **Cys322(C)** | **-6.3** | **2-B40** |  |
| **Cys322(A,C)**  **Gly323(A.C)** | **-** | **-** | **-6.1** | **iBuCHO** |  |

**Table S6. Binding affinity, hydrogen bonding, and hydrophobic interactions of ligands to the SKCGS sequence in chains B, D, and F.**

| **Hydrophobic interactions**  **Amino acid (chain)** | **Hydrogen Bonding** | | **Affinity**  **(kcal/mol)** | **Crude spices and their constituents** | |
| --- | --- | --- | --- | --- | --- |
|  | **Distance (Å)** | **Amino acid (chain)** |  |  |  |
| **Cys 322 (B)** | **2.51** | **Lys 321 (F)** | **-6.7** | **CNMA** | **CN** |
|  | **2.66** | **Lys 321 (B)** |  |  |  |
| **Lys 321 (D)**  **Cys 322 (B)**  **Gly 323 (B,F)** | **3.22** | **Lys 321 (B)** | **-6.2** | **Cin** |  |
| **Lys 321 (B,D)**  **Cys 322 (B)** | **3.19** | **Lys 321 (F)** | **-6.8** | **α-TA** | **Car** |
| **Lys 321 (B)**  **Cys 322 (B,D)**  **Gly 323 (B)** | **-** | **-** | **-7** | **CNL** |  |
| **Lys 321 (B)**  **Cys 322 (B)** | **2.94** | **Lys 321 (F)** | **-6.7** | **PEA** | **ROSE** |
| **Lys 321 (B,D)**  **Cys 322 (B,D)**  **Gly 323 (D)** | **2.83** | **Lys 321 (F)** | **-6.8** | **Cit** |  |
| **Lys 321 (D,F)**  **Cys 322 (B,D,F)** | **2.92** | **Lys 321 (B)** | **-6.7** | **Gra** |  |
| **Lys 321 (D)**  **Cys 322 (B,D)** | **2.74** | **Lys 321 (B)** | **-6.1** | **Safr** | **Saf** |
| **Lys 321 (B)**  **Cys 322 (B)** | **2.86** | **Lys 321 (F)** | **-6.2** | **2-B40** |  |
| **Lys 321 (B)** | **2.79** | **Lys 321 (F)** | **-6.8** | **iBuCHO** |  |


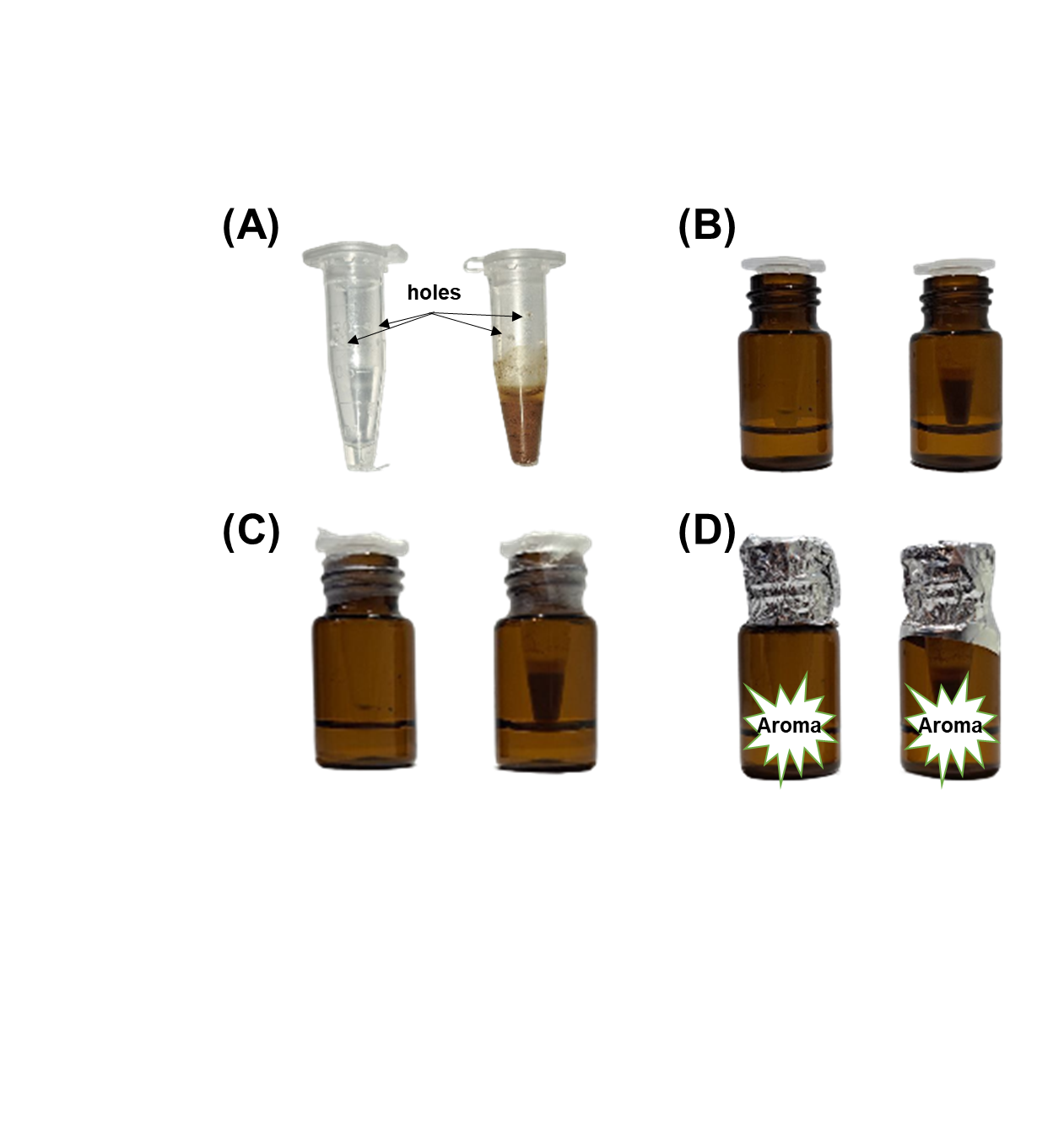


**Figure S1. The aroma-based incubation setup.** (A) Microtubes containing pure active constituents and common crude spices, featuring small holes for dispersing the volatile phytoaromatic compounds. (B-D) The insertion of microtubes into bottles, followed by sealing their lids with parafilm and subsequently aluminum film, respectively.


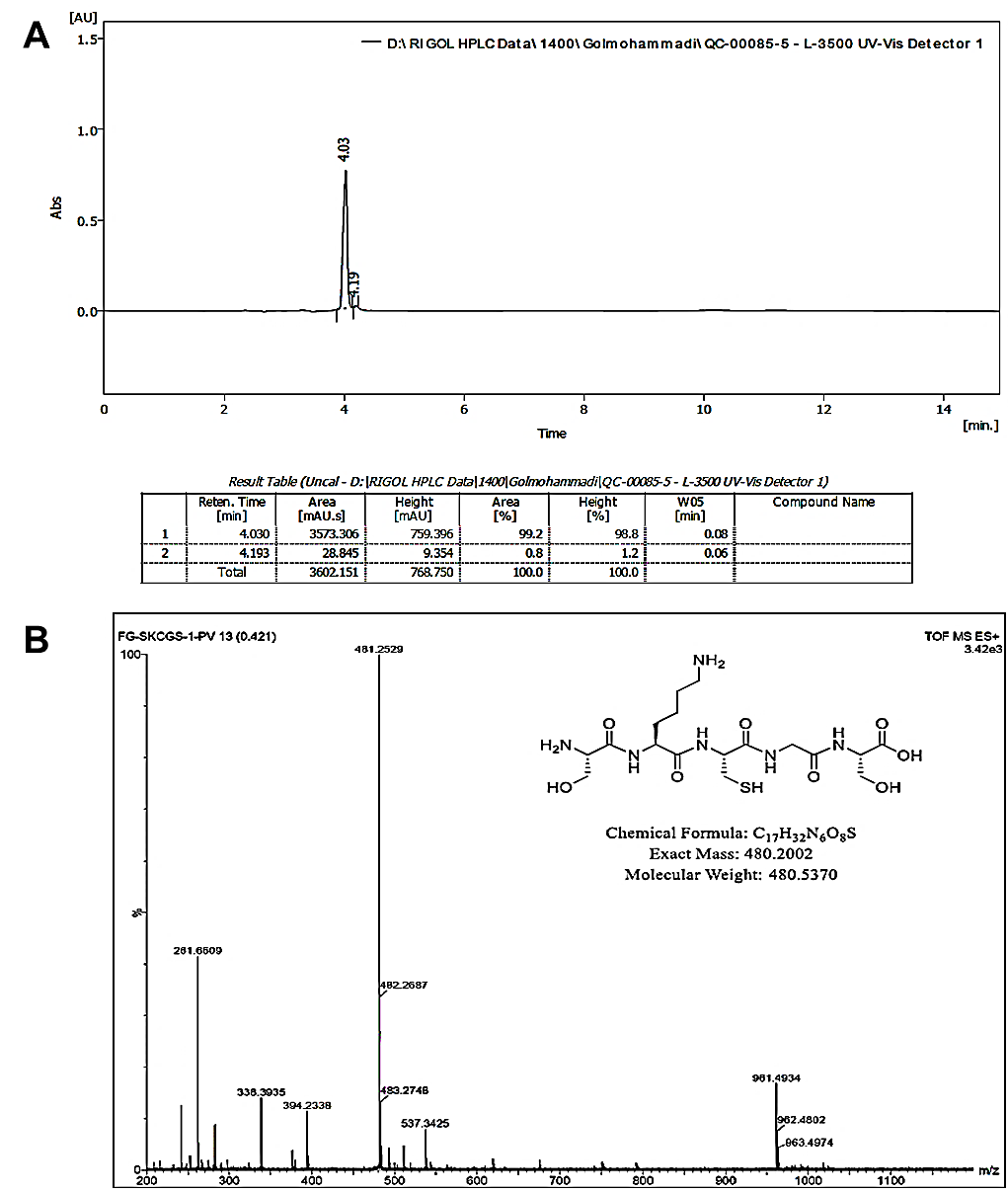


**Figure S2. Data revealing the purity and molecular weight of the SKCGS peptide with (A) MALDI-TOF-MS and (B) HPLC.**


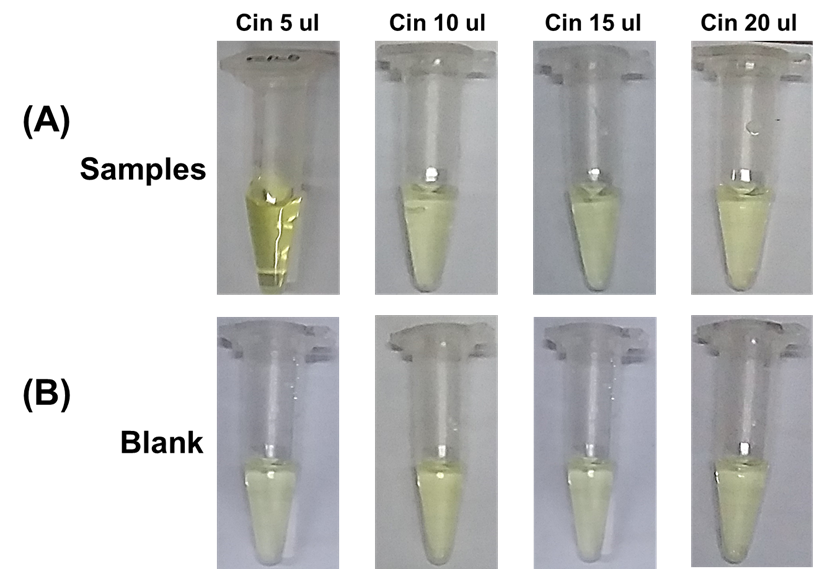


**Figure S3**. **The reaction of Cin solutions at various volumes with (A) samples containing the SKCGS peptide solution, and (B) blank controls.**

**
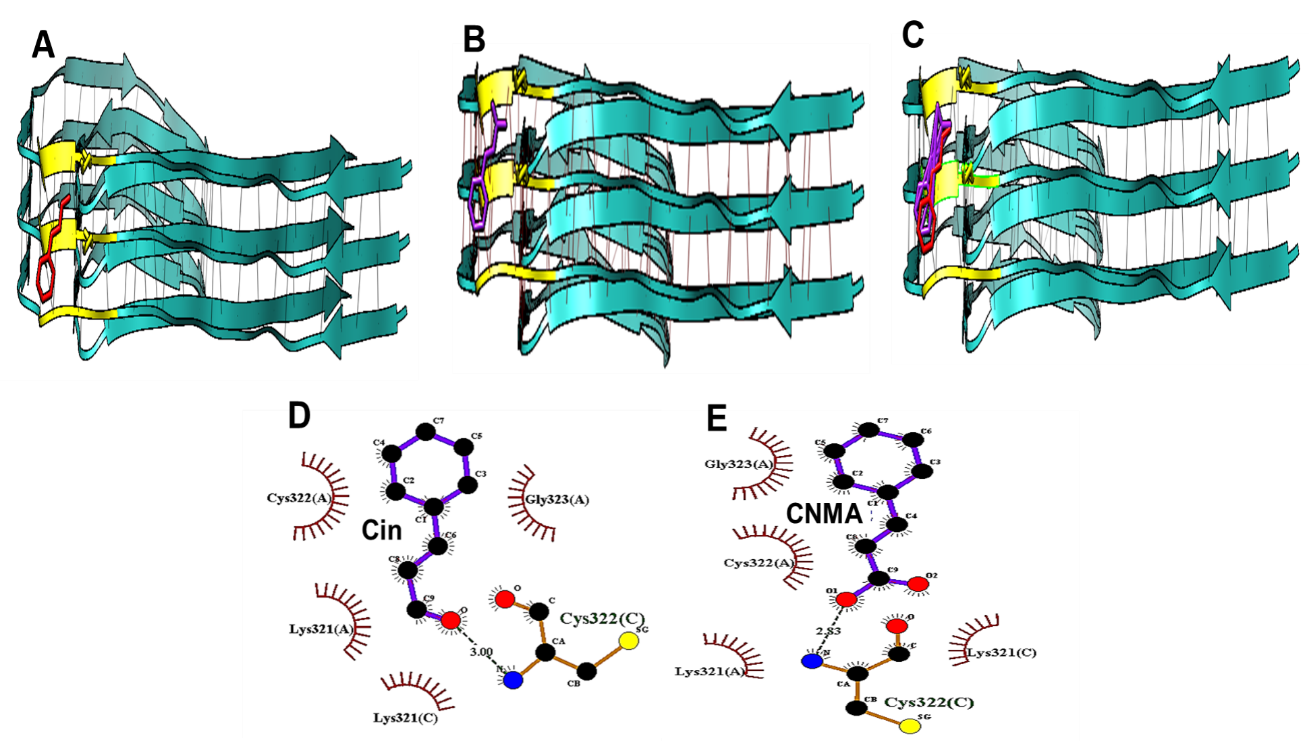
**

**Figure S4. DockThor potential binding modes of active constituents of CN to the SKCGS peptide in chains A, C and E.** (A and D) Shows Cin binding to the SKCGS peptide. (B and E) Shows CNMA binding to the SKCGS peptide. (C) Shows the Cin and CNMA superposed binding to the SKCGS peptide. The figure was made using the Chimera and Ligplot softwares.


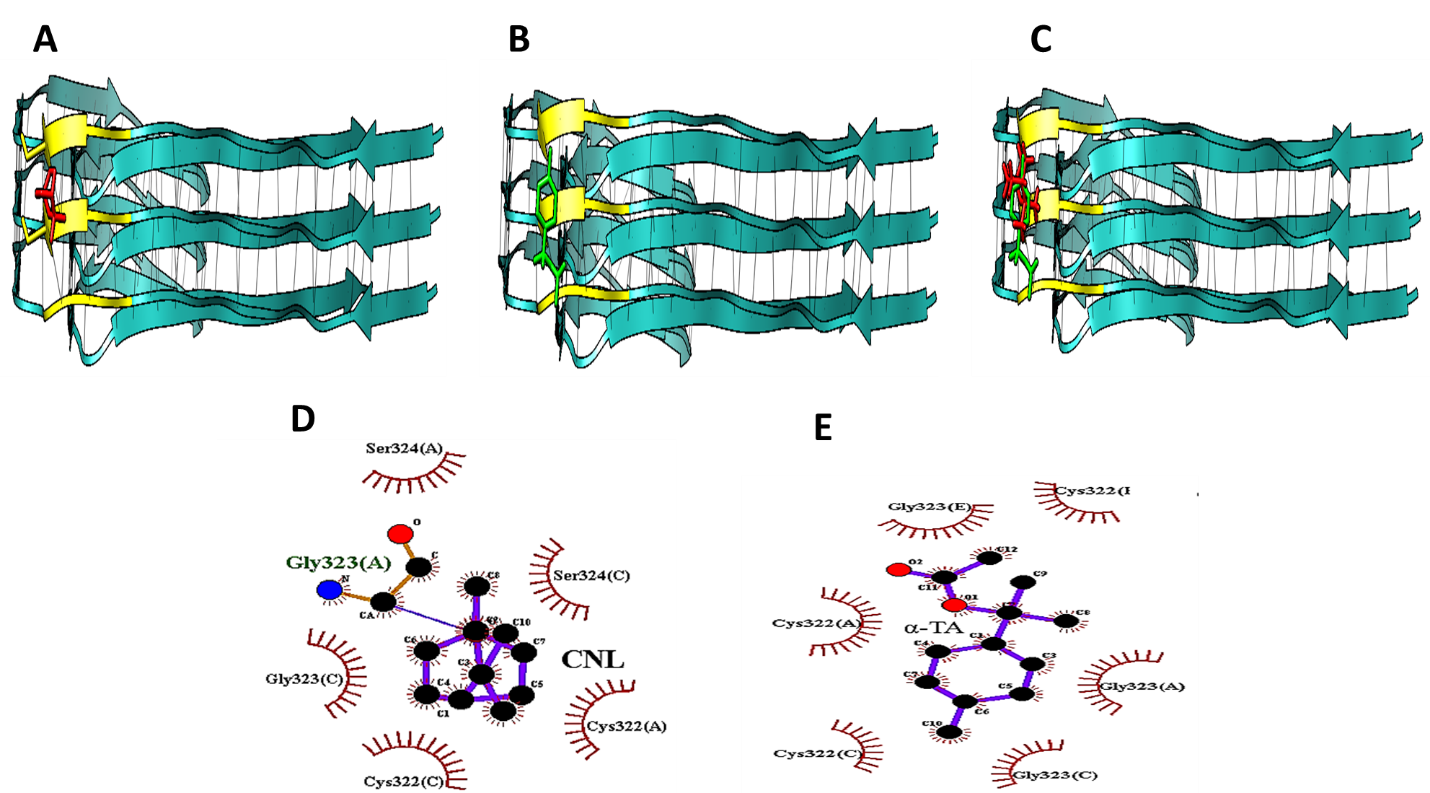


**Figure S5. DockThor potential binding modes of active constituents of Car to the SKCGS peptide in chains A, C and E.** (A and D) Shows CNL binding to the SKCGS peptide. (B and E) Shows α-TA binding to the SKCGS peptide. (C) Shows the CNL and α-TA superposed binding to the SKCGS peptide. The figure was made using the Chimera and Ligplot softwares.


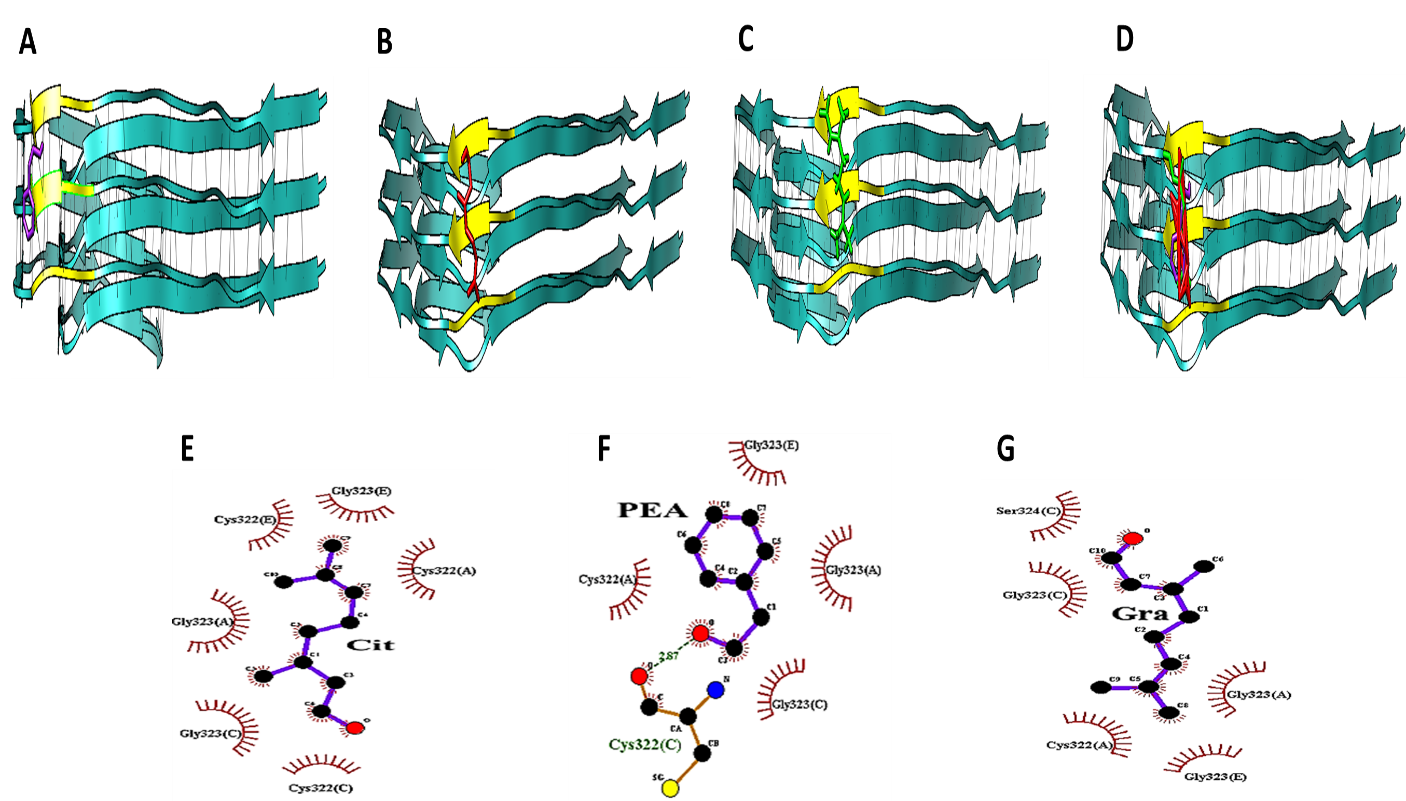


**Figure S6. DockThor potential binding modes of active constituents of Rose to the SKCGS peptide in chains A, C and E.** (A and E) Shows Cit binding to the SKCGS peptide. (B and F) Shows PEA binding to the SKCGS peptide. (C and G) Shows Gra binding to the SKCGS peptide. (D) Shows the Cit, PEA and Gra superposed binding to the SKCGS peptide. The figure was made using the Chimera and Ligplot softwares.


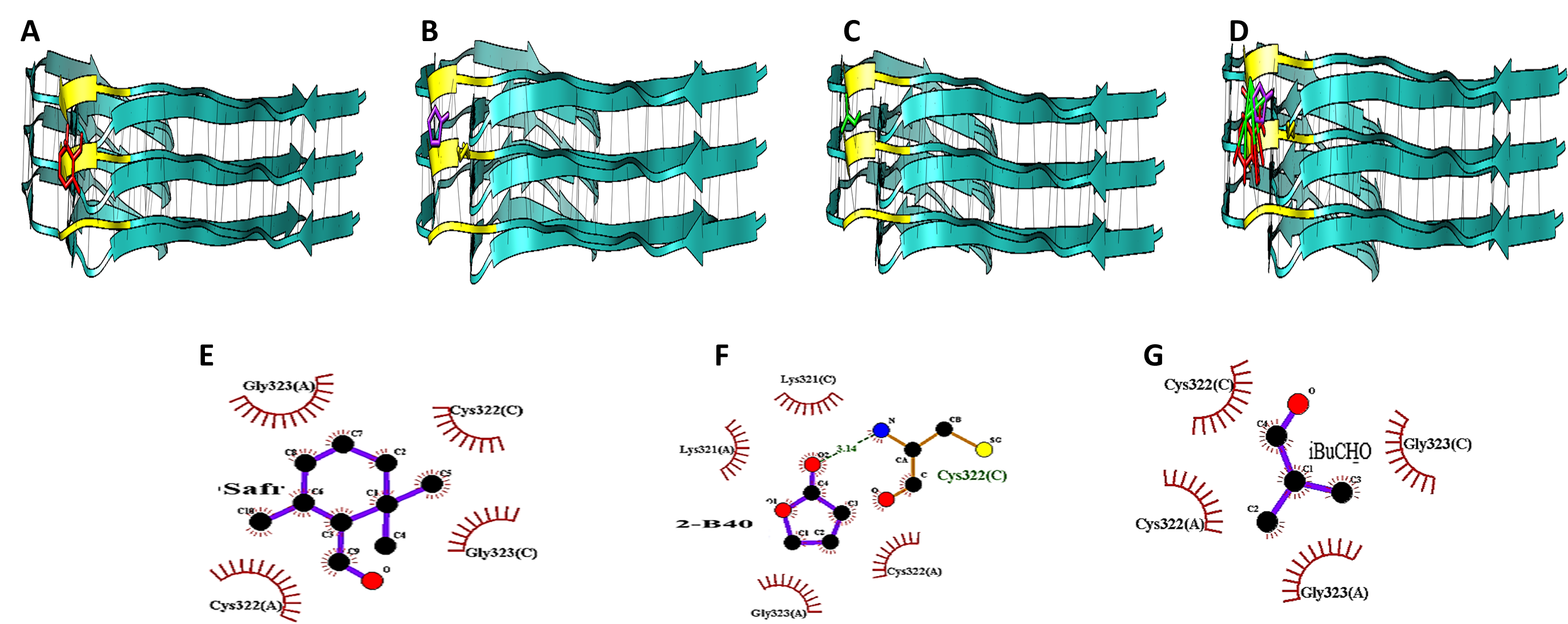


**Figure S7. DockThor potential binding modes of active constituents of Saf to the SKCGS peptide in chains A, C and E.** (A and E) Shows Safr binding to the SKCGS peptide. (B and F) Shows 2-B4O binding to the SKCGS peptide. (C and G) Shows iBuCHO binding to the SKCGS peptide. (D) Shows the Safr, 2-B40 and iBuCHO superposed binding to the SKCGS peptide. The figure was made using the Chimera and Ligplot softwares.


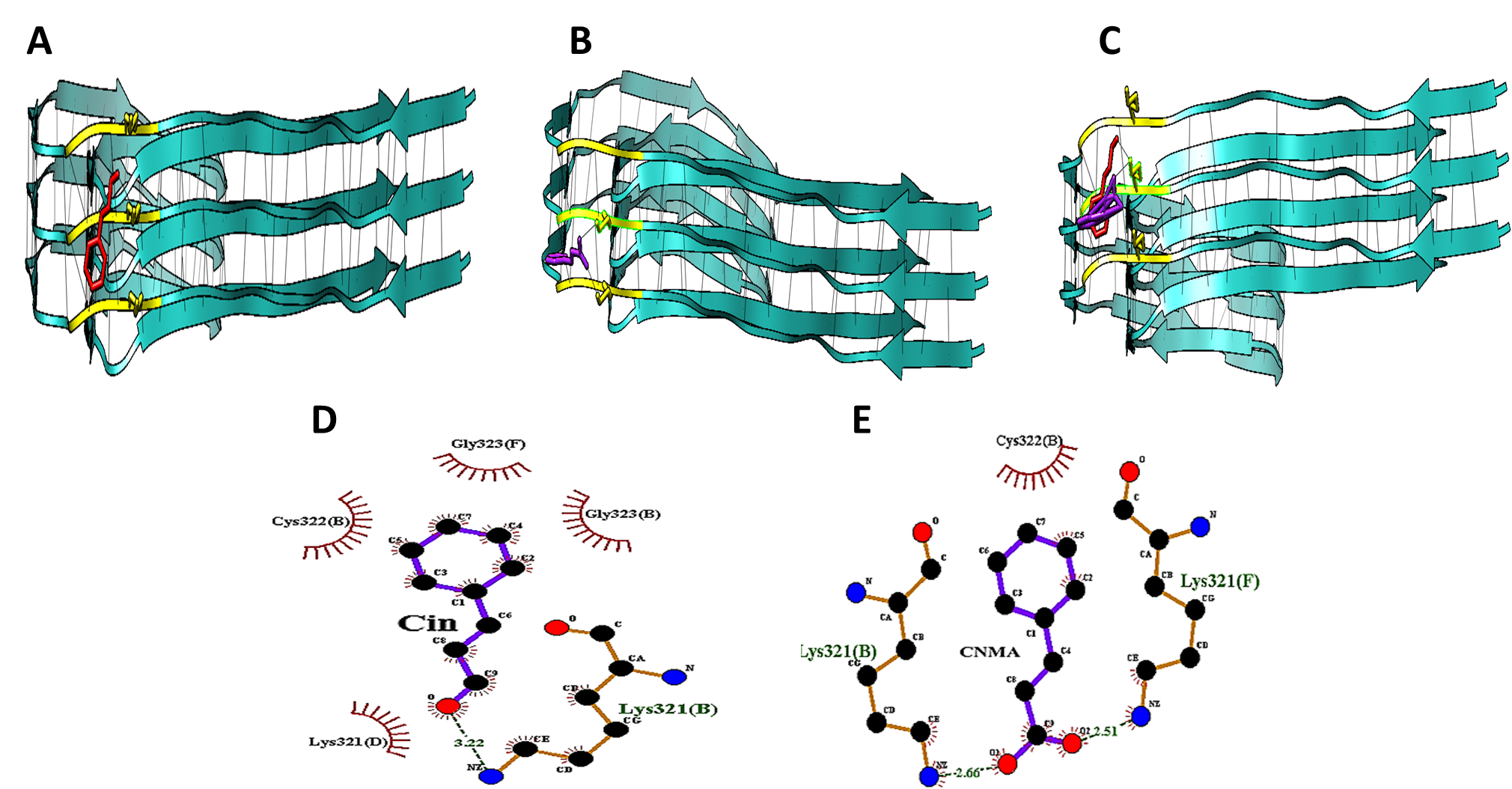


**Figure S8. DockThor potential binding modes of active constituents of CN to the SKCGS peptide in chains B, D and F.** (A and D) Shows Cin binding to the SKCGS peptide. (B and E) Shows CNMA binding to the SKCGS peptide. (C) Shows the Cin and CNMA superposed binding to the SKCGS peptide. The figure was made using the Chimera and Ligplot softwares.


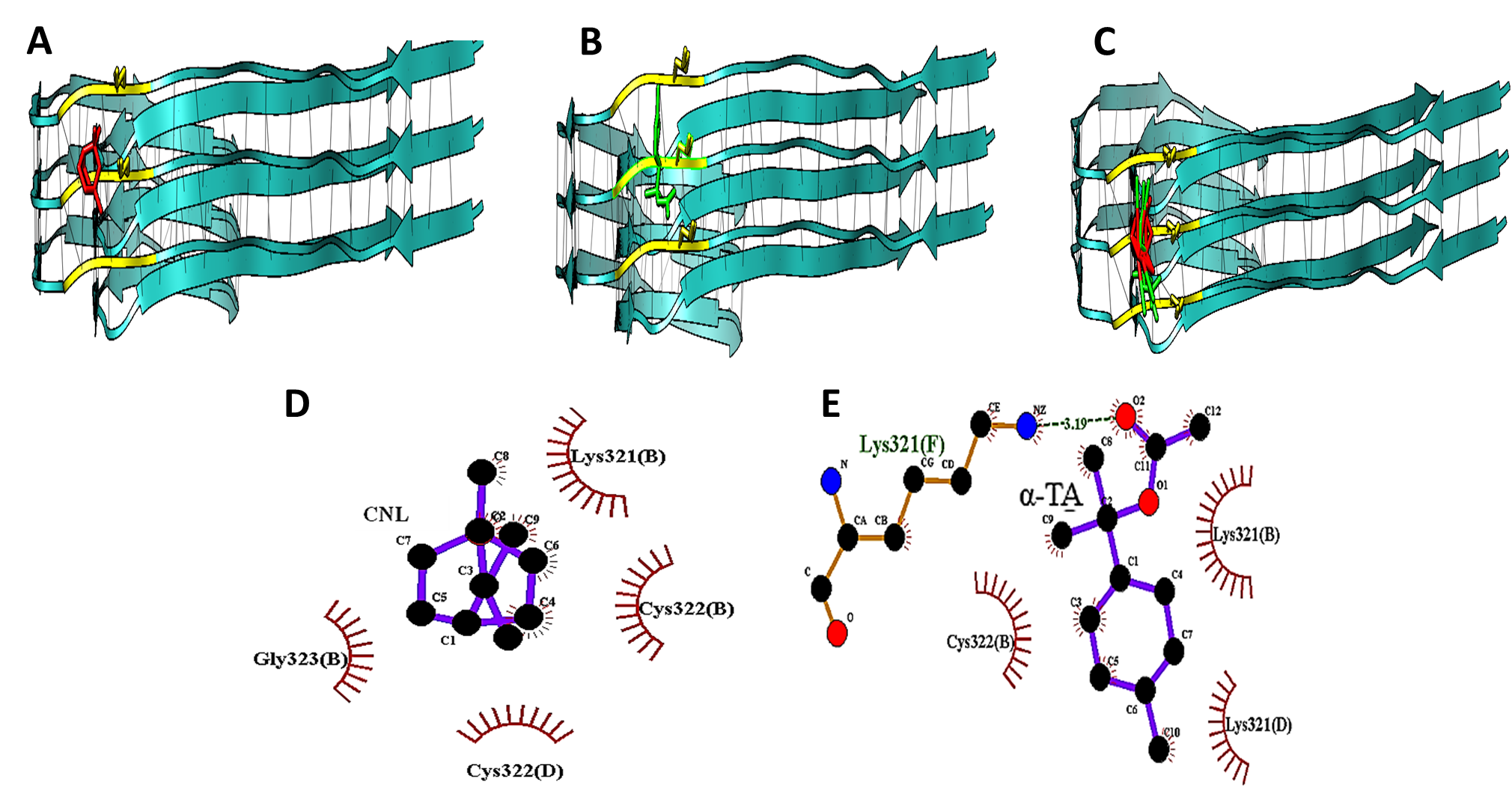


**Figure S9. DockThor potential binding modes of active constituents of Car to the SKCGS peptide in chains B, D and F.** (A and D) Shows CNL binding to the SKCGS peptide. (B and E) Shows α-TA binding to the SKCGS peptide. (C) Shows the CNL and α-TA superposed binding to the SKCGS peptide. The figure was made using the Chimera and Ligplot softwares.


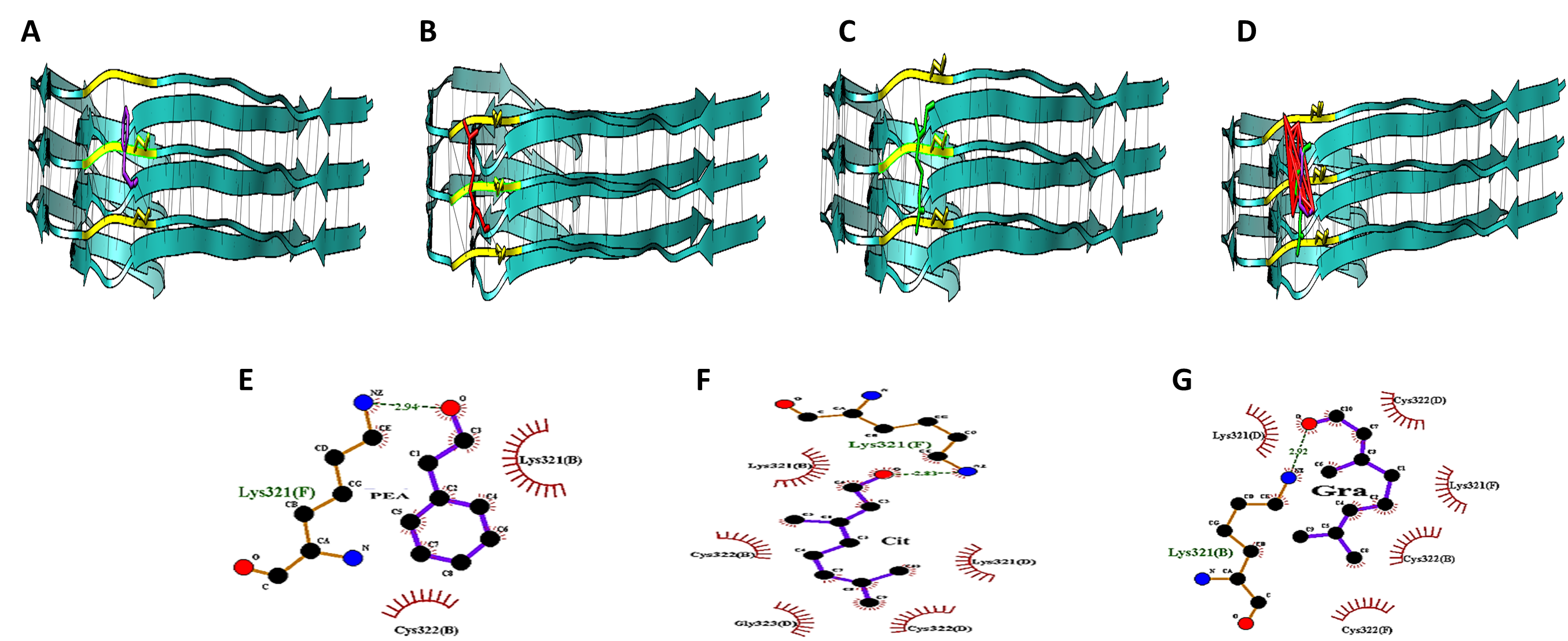


**Figure S10. DockThor potential binding modes of active constituents of Rose to the SKCGS peptide in chains B, D and F.** (A and E) Shows PEA binding to the SKCGS peptide. (B and F) Shows Cit binding to the SKCGS peptide. (C and G) Shows Gra binding to the SKCGS peptide. (D) Shows the PEA, Cit and Gra superposed binding to the SKCGS peptide. The figure was made using the Chimera and Ligplot softwares.


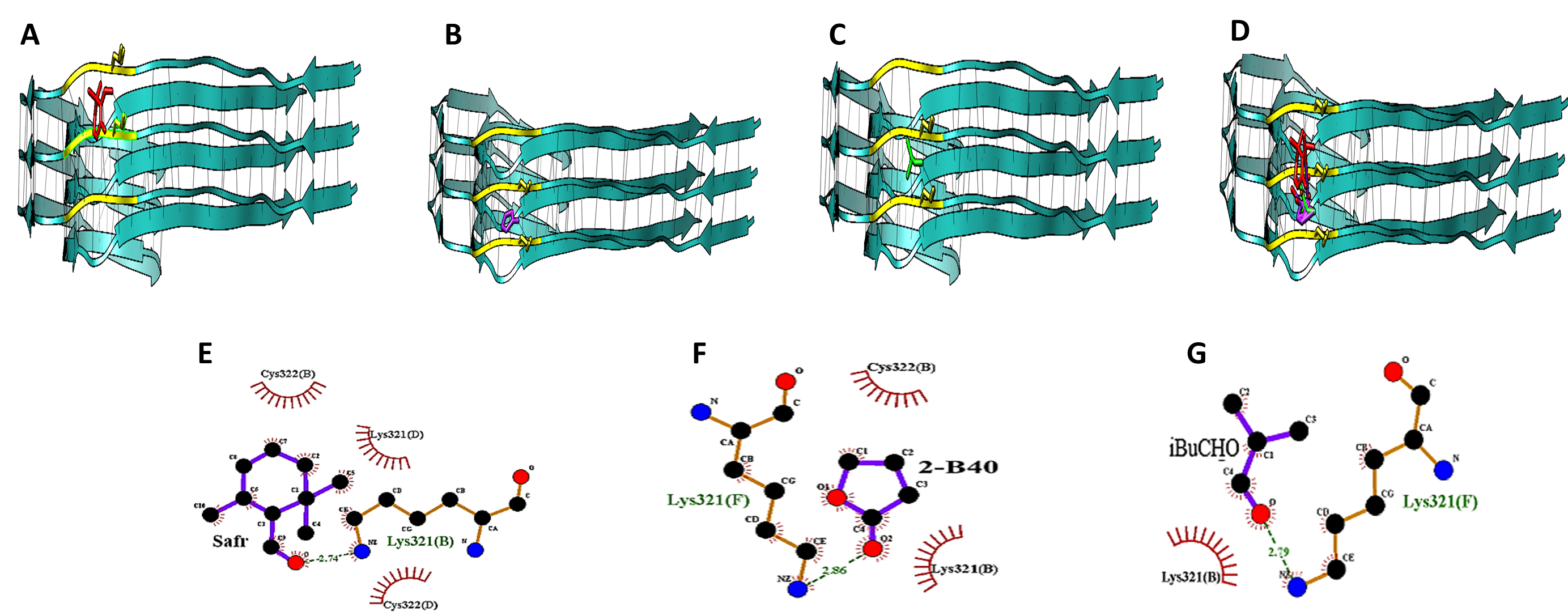


**Figure S11. DockThor potential binding modes of active constituents of Saf to the SKCGS peptide in chains B, D and F.** (A and E) Shows Safr binding to the SKCGS peptide. (B and F) Shows 2-B4O binding to the SKCGS peptide. (C and G) Shows iBuCHO binding to the SKCGS peptide. (D) Shows the Safr, 2-B40 and iBuCHO superposed binding to the SKCGS peptide. The figure was made using the Chimera and Ligplot softwares.


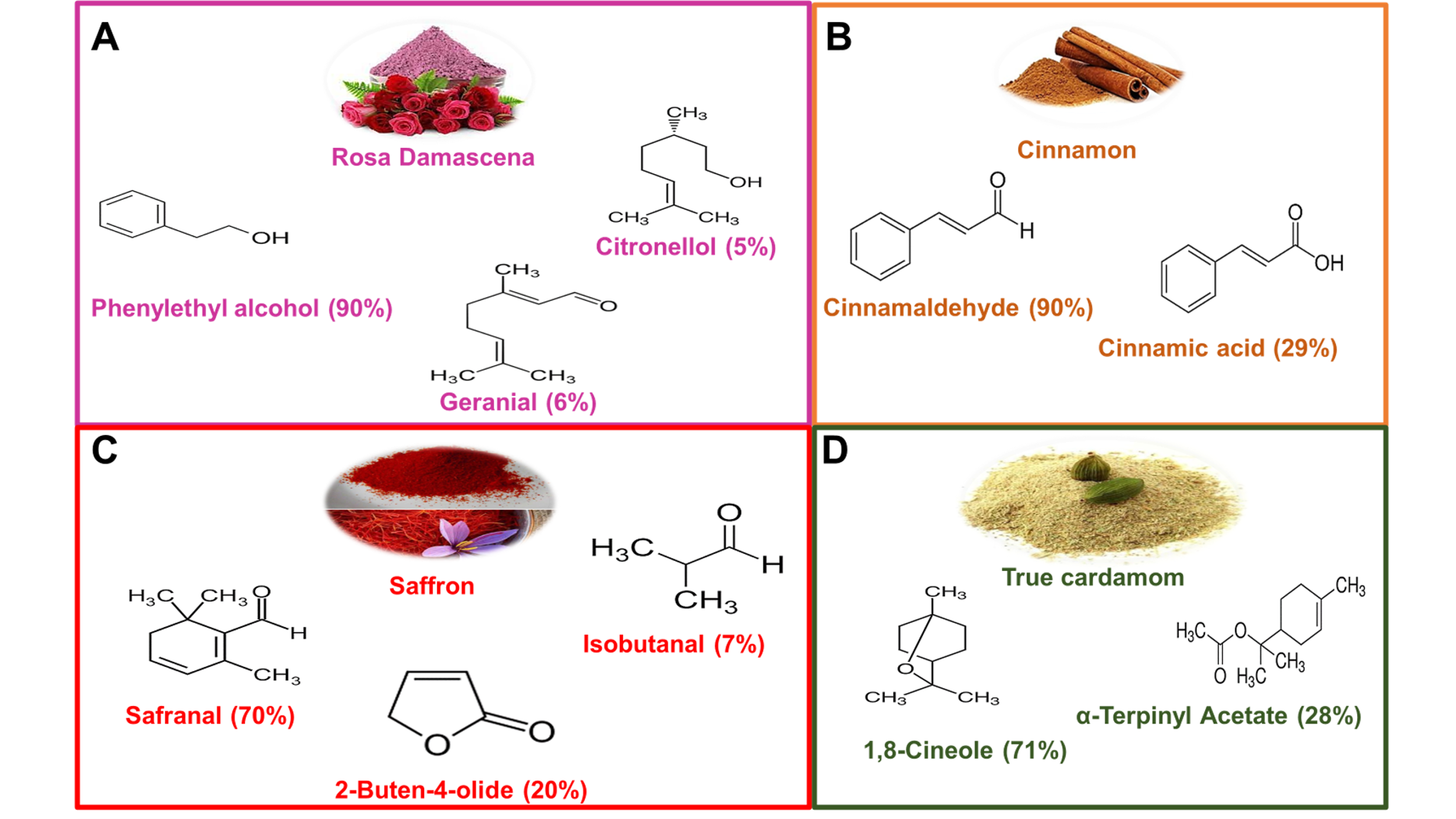


**Figure S12. Common crude spices and their main ingredients.** (A) Rose, (B) Cinnamon, (C) Saffron and (D) True cardamom [21,47–54].
